# Locust-Derived Biohybrid Muscle Actuators for Low-Power Explosive Jumping

**DOI:** 10.34133/research.0943

**Published:** 2025-10-21

**Authors:** Peng Liu, Yao Li, Songsong Ma, Yunhao Si, Bing Li

**Affiliations:** ^1^ School of Robotics and Advanced Manufacture, Harbin Institute of Technology, Shenzhen 518055, China.; ^2^ State Key Laboratory of Robotics and Systems, Harbin Institute of Technology, Harbin, China.; ^3^ Guangdong Provincial Key Laboratory of Intelligent Morphing Mechanisms and Adaptive Robots, Harbin Institute of Technology, Shenzhen, China.; ^4^ Key University Laboratory of Mechanism & Machine Theory and Intelligent Unmanned Systems of Guangdong, Harbin Institute of Technology, Shenzhen, China.; ^5^ School of Mechanical and Electrical Engineering, the Shenzhen Polytechnic University, Shenzhen 518055, China.

## Abstract

A critical challenge for jumping microrobots is achieving a compact actuator with a high energy output as traditional elastic actuators are inherently bulky. The integration of biological materials with artificial systems to realize biohybrid muscle actuators is a promising approach. However, previous attempts utilizing the entire organism have been hampered by the unpredictability of the native nervous system, and actuators integrating cultivated or extracted muscle tissues have so far been unable to achieve a sufficiently explosive output capacity for jumping. Here, discarded locust hindlegs are repurposed into explosive biohybrid muscle actuators that are synergistically integrated with an artificial robotic system. The resulting biohybrid locust is only 2 g in weight and is precisely controlled through electrical stimulation to achieve dynamic leaps of up to 18 times its body length and 7 times its body height, which outperforms most synthetic counterparts. The design exhibits 2 key functional advances: on the one hand, the actuator requires an ultralow-power input of only 0.03 mW via the optimization of stimulation protocols; on the other hand, the actuator rapidly releases kinetic energy, enabling the artificial robotic system to perform long-distance jumps. This paper presents an experimental validation and biomechanical analysis on the biohybrid locust to demonstrate how our strategy unlocks sustainable and high-performance actuation for microrobots. This work pioneers a roadmap for the next generation of biohybrid robots that merge ecological sustainability with engineering excellence.

## Introduction

The development of functional materials for miniaturized actuation systems is a critical challenge for applications such as exploration and emergency response in confined environments. While elastic energy storage mechanisms such as springs have been widely employed to create jumping actuators [1], such systems face inherent scaling limitations that reduce their efficiency when miniaturized: they struggle to accumulate sufficient energy for effective jumping, and their reset mechanisms often introduce additional energy losses [2]. Some scholars have used piezoelectric [3] or dielectric [4] materials to fabricate low-power actuators, but these actuators cannot be applied to jumping. In biology, jumping has proven to be an efficient mode of locomotion for small animals. Thus, some researchers have focused on leveraging biological materials to create ultracompact actuators. Various studies have explored directly converting organisms such as insects [5] or vertebrates [6] into bio-robots [7–9]. However, the movement of these bio-robots is constrained by the unpredictability of the organism’s nervous system. Recent advances have seen the development of biohybrid muscle actuators, which integrate biological tissues with artificial actuators. Some scholars have constructed artificially cultivated muscle tissues into swimmers [10], crawlers [11], or grippers [12] while others have directly extracted muscle tissues from living organisms to fabricate kicking actuators [13] or pumps [14]. However, these actuators still lack sufficient explosive output capacity for jumping, and they cannot work without a culture solution [15].

This study addressed these challenges to develop an ultracompact and high-power-density biohybrid muscle actuator through a sustainable and innovative repurposing of a biological waste resource: discarded locust hindlegs. Locusts are talented jumpers owing to their powerful hindlegs, which helps them avoid predators. However, the hindlegs become long and outstretched as they jump and may be caught in emergency situations. In such cases, the locust will break off the hindleg as a last-ditch effort to escape. Such discarded hindlegs can remain active in air for several hours and respond when an appropriate electrical stimulus is applied. Thus, discarded locust hindlegs were collected and transformed into biohybrid muscle actuators capable of producing an explosive force (Movies S1 and S2). The biohybrid muscle actuators were then incorporated into a microrobot with an artificial joint. The resulting biohybrid locust has a mass of only 2 g and exhibits locust-like jumping abilities (Movies S3 to S5) and self-righting capabilities upon landing for successive jumping owing to the synergistic manipulation of biological and artificial materials (Movies S6 and S7). This study elucidated the mechanisms by which biohybrid muscle actuators can achieve low power consumption and compact dimensions for precise jumping control.

## Results

### Evaluation of the actuator performance

#### Electrical stimulation

**Fig. 1.**
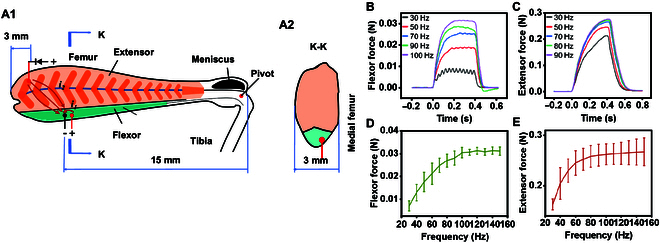
Electrical stimulation of the isolated locust hindlegs (*N* = 5 hindlegs, *n* = 25 trials). (A) Schematic diagram of the locust hindleg where the extensor muscle is in light orange and the flexor is in green: (A1) side view and (A2) cross-section at K–K. The dark orange indicates the pinnation of the extensor muscle. (B) Flexor force and (C) extensor force in the temporal domain according to the signal frequency of the electrical stimulus. (D) Flexor force and (E) extensor force in the frequency domain. The error bars represent the mean ± standard deviation.

Electrical stimulation via pulsed signals is commonly used to induce contraction in a muscle [16]. A low signal frequency elicits a twitch contraction whereas a high signal frequency elicits a tonic contraction because of insufficient relaxation between successive signals, which leads to the summation effect. Consequently, the force of a muscle can be regulated by adjusting the signal frequency. Figure 1A illustrates the effective electrode implantation sites in the isolated locust hindlegs. Electrical stimulation successfully induced contractions of the flexor and extensor muscles of the isolated locust hindlegs (Movie S1). Figure 1B and C and show the forces of the flexor and extensor muscles, respectively, in the temporal domain according to the signal frequency of the electrical stimulus. The flexor force reached its peak value ~0.2 s after stimulation, and a higher signal frequency generally increased the flexor force and stability. For example, at a signal frequency of 30 Hz, the flexor force reached 8 mN with a fluctuation of 2 mN; at a signal frequency of 100 Hz, the flexor force reached 31 mN with a fluctuation of only 0.5 mN. In contrast, the extensor force increased with the signal frequency up to 70 Hz and then did not increase further. Because the flexor muscle generated a steady force, it could be finely controlled by modulating the signal frequency. However, the force generated by the extensor muscle increased continuously during stimulation, which indicated that it can be regulated by keeping the signal frequency constant and instead adjusting the duration of the electrical stimulation. Figure 1D and E show the forces of the flexor and extensor muscles, respectively, in the frequency domain. The extensor reached a force of 258 ± 21 mN at 80 Hz, which was over 8 times greater than the flexor force. This difference can be attributed to the flexor muscle having a smaller mass (0.015 g) than the extensor muscle (0.08 g). The muscle fibers also differed in appearance (Movie S1). The extensor muscle fibers had a length of 2.5 to 4.3 mm with a pennate distribution and a maximum cross-sectional area of 16 mm^2^. The flexor muscle fibers had a length of 7 mm with a parallel distribution and a cross-sectional area of only 2 mm^2^. The distribution of muscle fibers had a notable impact on the relaxation time. In particular, the flexor muscle needed ~0.08 s to revert to a relaxed state following the termination of electrical stimulation while the extensor muscle took ~0.25 s. Further results from tests on the isolated locust hindlegs are presented in the Supplementary Materials (Note S3).

#### Kinematic performance

**Fig. 2.**
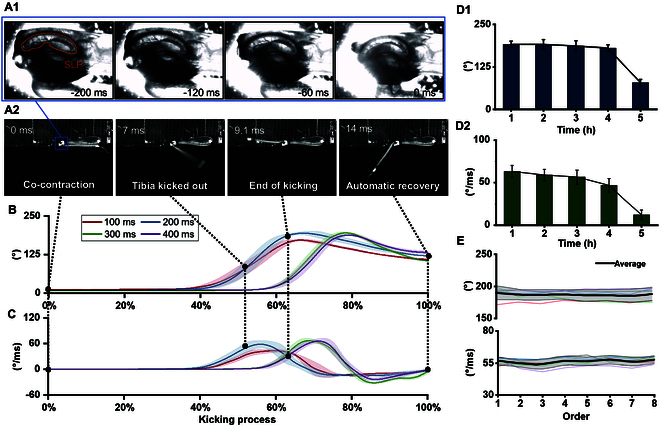
Performance of biohybrid muscle actuators. (A) Co-contraction process in the biohybrid muscle actuator: (A1) close-up view of the joint during co-contraction and (A2) the kicking process of the biohybrid muscle actuator after co-contraction. The termination of the co-contraction process released the actuator and resulted in a kick. (B) Kicking angle and (C) angular velocity of actuators according to the co-contraction time (*N* = 20 hindlegs, *n* = 28 trials). The solid line represents the curve after averaging, and the shaded area represents the standard deviation. The kicking angle (D1) and kicking angular velocity (D2) responses of the actuators according to the separation time (*N* = 5 hindlegs, *n* = 5 trials). Points on the connecting line are the mean values. (E) Fatigue curves for 8 successive kicks (*N* = 5 hindlegs, *n* = 5 trials). The thin lines indicate the fatigue curves from different samples, the thick lines indicate the mean values, and the light gray areas represent the standard deviations.

As shown in Fig. 2A, locusts jump by co-contracting flexor and extensor muscles to accumulate energy in the semi-lunar process (SLP) [16] and then rapidly releasing the energy to generate an explosive force. In this study, a pulse sequence of electrical stimulation was designed that effectively initiated the co-contraction to induce an instant kick by the isolated locust hindleg (Movie S2). When the maximum angle was reached, the tibia naturally returned to a neutral position because of the inherent rebound properties of the flexor and extensor muscles. Under unloaded conditions, the internal retracting moment was ~0.48 mN·mm, and the abducting moment was ~1.34 mN·mm. The kicking process of the biohybrid muscle actuators was directly influenced by the co-contraction time and was positively correlated with the elastic deformation of the SLP. Figure 2B shows the kicking angle of the biohybrid muscle actuators according to the co-contraction time. At co-contraction times of 100 to 200 ms, the biohybrid muscle actuator reached the maximum kicking angle at ~65% of the kicking process. At co-contraction times of 300 to 400 ms, the biohybrid muscle actuators reached the maximum kicking angle at ~80% of the kicking process, which can be attributed to the longer-term effects of the residual charge. Co-contraction times of 200 to 400 ms all yielded relatively large kicking angles of ~190°, which is greater than the natural kicking angle of ~180° [16]. Figure 2C shows the angular velocity of the biohybrid muscle actuator according to the co-contraction time. At co-contraction times of 100 to 200 ms, the peak angular velocity occurred at ~57% of the kicking process. At co-contraction times of 300 to 400 ms, the peak angular velocity was reached at ~70% of the kicking process. The kinematic parameters of the biohybrid muscle actuators became inconsistent when the co-contraction time exceeded 400 ms (Fig. S7). At co-contraction times of 200 to 400 ms, the angular velocity was ~70°·ms^−1^, which approximates the natural angular velocity [14]. Notably, the kicks of the biohybrid muscle actuators were weaker than those of the locust hindlegs in vivo, which have a kicking angle of ~200° and an angular velocity of ~95°·ms^−1^.

Most biohybrid muscle actuators created through tissue engineering are limited in their ability to generate sufficient force to produce an explosive output. For example, the output of an earthworm muscle actuator is merely 9.33 mN [14]. In addition, cultivated muscle tissues can be used only in specific nutrient solutions and have a short effective duration in the working environment [10]. In contrast, the locust hindlegs were used to obtain biohybrid muscle actuators with a greatly enhanced explosive output. The biohybrid muscle actuators were naturally encapsulated by the locust’s exoskeleton, which allowed them to retain their kicking performance for longer. As shown in Fig. 2D, even after 4 h of separation from the locust body, the biohybrid muscle actuators retained a kicking angle and angular velocity of ~180° and ~50°·ms^−1^, respectively, which indicates that they maintained >80% of their original performance. However, both the kicking angle and angular velocity began to decay with further separation time. This performance decay primarily results from physiological limitations: the severed end of the locust hindleg continuously lost fluid, and after ~4 h, the reduced fluid volume disrupted the metabolic environment and ionic balance of the muscles, which ultimately degraded the mechanical performance. To address this limitation, biocompatible encapsulation materials are needed to form a protective layer at the incision site [13] and thereby slow hemolymph loss rates to extend the functional duration of the biohybrid muscle actuators.

The fatigue characteristics of the biohybrid muscle actuators exhibited significant individual differences. As shown in Fig. 2E, they maintained a normal kicking performance for the first 8 successive kicks with a kicking angle of ~190° and an angular velocity of ~60°·ms^−1^ by the eighth kick. Further kicks resulted in individual differences with some biohybrid muscle actuators kicking less than 15 times while one kicked 47 times (Fig. S7). The amount of energy in the biohybrid muscle actuators may have contributed to the difference in kick counts. The input power for a pair of biohybrid muscle actuators was ~0.03 mW (Fig. S8), which is markedly lower than the power required for artificial miniature robots (100 to 1,000 mW) [17], and even more pronounced when compared with miniature jumping robots, whose reported power consumption is as high as 1,000 to 4,000 mW [18–21]. Actually, in the biohybrid system, the external electrical signal functions merely as a trigger, while the actual kinetic energy released during kicking originates from the biochemical energy stored within the muscle tissue. In system efficiency assessments, only externally supplied energy is typically considered as input [1,19], as this reflects the actual energy costs incurred by the engineering platform. Based on this definition, biohybrid actuators demonstrate extremely high energy conversion efficiency compared to purely artificial systems.

### Evaluation of the robot performance

#### Forward jump

**Fig. 3.**
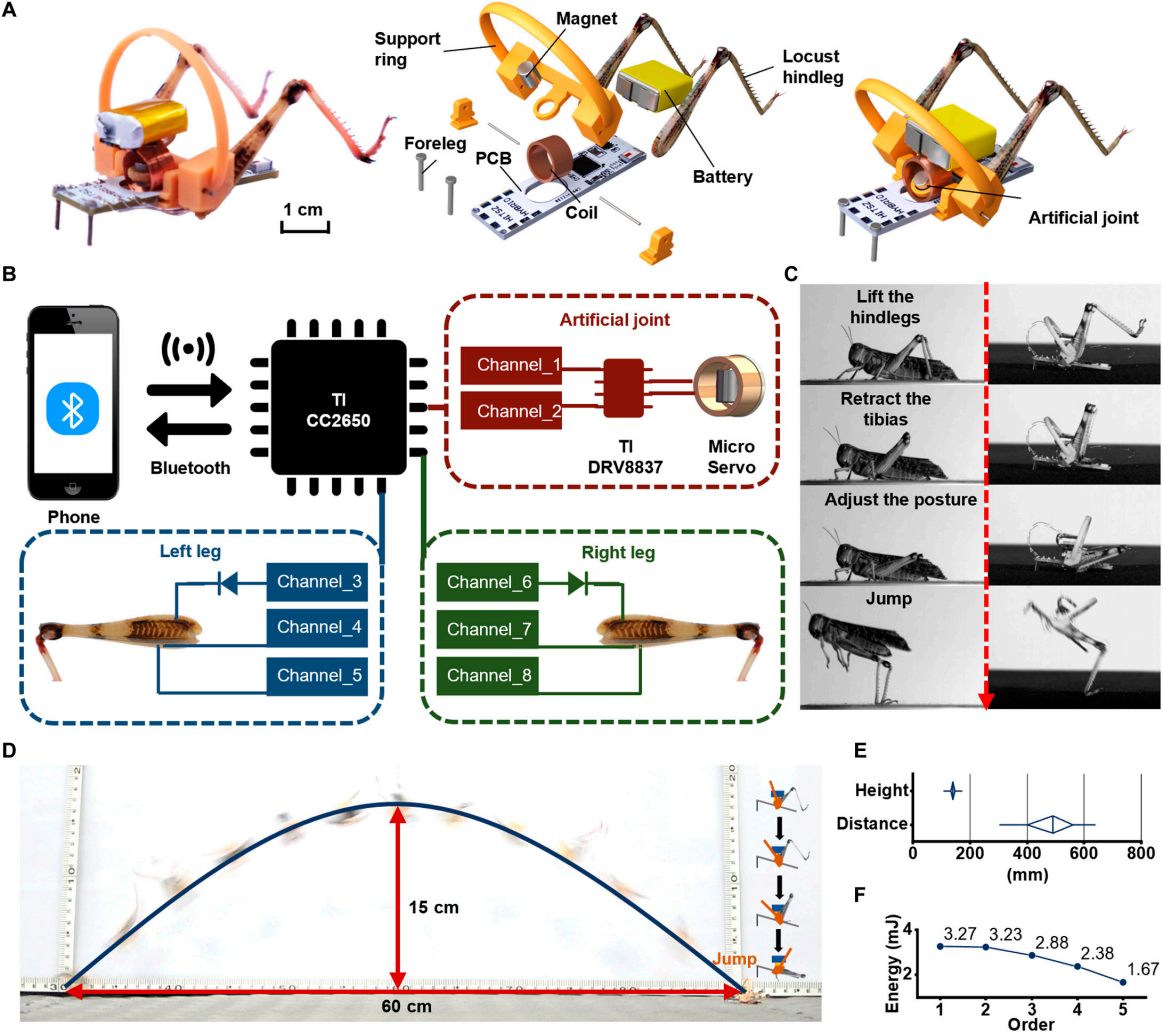
Forward jumps of the biohybrid locusts. (A) Picture and rendering of the biohybrid locust. (B) Control schematic. (C) Jumping sequences of the locust and biohybrid locust. (D) Jump trajectory. (E) Statistical plots of the jumping distance and height (*N* = 13 robots, *n* = 13 trials), where boxes represent the first quartile and the third quartile and whiskers represent the maximum or the minimum values. (F) Decay in kinetic energy with successive jumps.

The biohybrid muscle actuator makes a powerful and precise motion that can be used to realize a jump. However, the natural forward jump of a locust requires not only the explosive kick of their hindlegs but also the auxiliary movements of other joints. The biohybrid locust was created by integrating 2 biohybrid muscle actuators with an artificial joint to facilitate a successful forward jump (Movie S3). Figure 3A shows the biohybrid locust. Figure 3B shows the integration of the biohybrid muscle actuators with the control system. Figure 3C compares the jumping sequence of the biohybrid locust with that of an actual locust. A printed circuit board (PCB) was tasked with receiving remote commands and subsequently activating both the biohybrid muscle actuators and artificial joint. Based on the natural movement of the locust, the jumping process was designed by coordinating several steps. First, the artificial joint lifted the biohybrid muscle actuators, whose flexor muscles retracted the tibia. The artificial joint was then used to adjust the position of the biohybrid locust precisely. Energy was accumulated through co-contraction, which culminated in a powerful kick that propelled the biohybrid locust into the air. Figure 3D illustrates the complete trajectory of a biohybrid locust achieving an impressive jump (Movie S4). As shown in Fig. 3E, the biohybrid locusts achieved a maximum jump distance of 637.9 mm and a maximum jump height of 170.7 mm. Previous studies on bio-robots controlled the jumps of live locusts by muscle or nerve stimulation [7,16]. However, the biohybrid locusts outperformed these bio-robots. The kicking mechanism of the biohybrid muscle actuators demonstrated remarkable stability albeit with a decline in performance as the number of successive jumps increased. As shown in Fig. 3F, the kinetic energy of the biohybrid locust was 3.27 mJ for the first jump, decreased slightly to 3.23 mJ for the second jump, decreased by ~10% to 2.88 mJ for the third jump, decreased by ~20% to 2.38 mJ for the fourth jump, and decreased by ~30% to 1.67 mJ for the fifth jump.

#### Steering jump

**Fig. 4.**
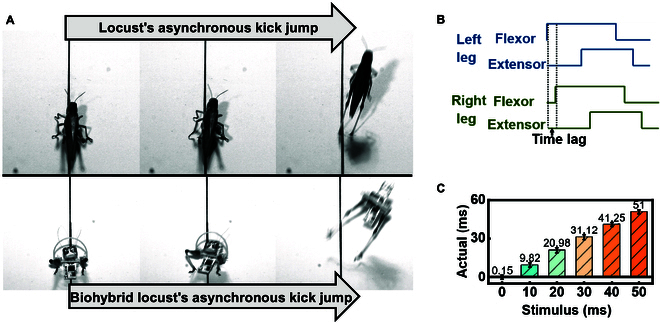
Steering jumps of the biohybrid locusts. (A) Comparison of steering jumps by a locust and biohybrid locust steer via asynchronous kicks. (B) Time lag for control of the biohybrid muscle actuators. High and low lines indicate when the muscles were excited and unexcited, respectively. (C) Actual time lag between the kick motions of both biohybrid muscle actuators at different time lags for the electrical stimulus (*N* = 12 hindlegs, *n* = 24 trials).

In addition to forward jumps, the biohybrid locust can also execute steering jumps. Locusts make steering jumps via asynchronous kicks of their hindlegs [22]. As shown in Fig. 4A, this steering jump can be mimicked by introducing a time lag between the motions of the 2 biohybrid muscle actuators (Movie S5). As shown in Fig. 4B, introducing a time lag between the control signals of the biohybrid muscle actuators resulted in a similar time lag in the resulting kicks (Movie S2). The isolated locust hindlegs exhibited a superior response agility than in vivo. As shown in Fig. 4C, the difference between the time lags of the electrical stimulus and kicking motion was as short as ~1.5 ms compared to ~5 ms in vivo [22]. The isolation of the neural signal may have aided precision, which would result in better and more flexible steering.

**Fig. 5.**
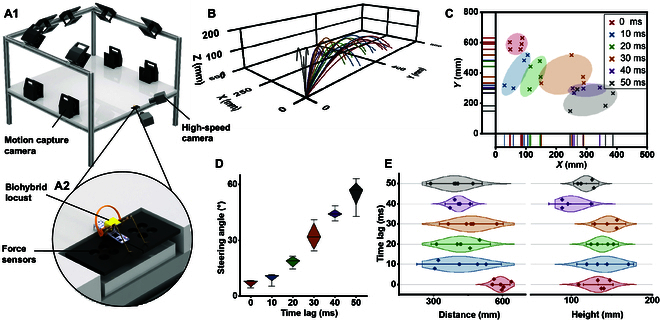
Steering jump control. (A1) Reconstructed image of the integrated jumping experiment platform, and (A2) a magnified view of the multipoint matrix force plate where a biohybrid locust prepares to jump. (B) Steering jump trajectory with different time lags (*N* = 25 robots, *n* = 25 trials). (C) Landing positions of steering jumps grouped by time lags. (D) Statistical plots of steering angles with different time lags, where boxes represent the first quartile and the third quartile and whiskers represent the maximum or the minimum values. (E) Statistical plots of distances and heights of steering jumps with different time lags, where shaded areas represent the probability density and whiskers represent the 95% confidence interval.

Different time lags affected the steering angles of the jump trajectories. The trajectories and landing positions were reconstructed, as shown in Fig. 5B and C, and the force profiles of the 2 biohybrid muscle actuators during a steering jump were obtained (Fig. S13B). Notable steering angle changes due to time lags were observed, as shown in Fig. 5D. The steering angle, which was defined as the deviation between the landing point and the *Y*-axis direction, increased from 4° to 8° at a time lag of 0 ms to 43° to 63° at a time lag of 50 ms. The asynchronous kicks used to steer the jump meant that one biohybrid muscle actuator kicked before the other, and the direction and magnitude of the force exerted by the second actuator were influenced by those of the first. Figure 5E compares the jump distances and jump heights according to the time lag between the 2 actuators. The jump distance was ~600 mm with no time lag (i.e., 0 ms) but decreased to ~420 mm at time lags of 10 to 50 ms. The jump height remained relatively constant at ~150 mm at time lags of 0 to 30 ms but dropped to ~100 mm at time lags of 40 to 50 ms. Thus, the jump distance was more sensitive to the time lag than the jump height. In brief, the biohybrid locust showcased remarkable control of its jumps via asynchronous kicks, which is a practical strategy for jumping microrobots to overcome obstacles.

#### Successive jumps

**Fig. 6.**
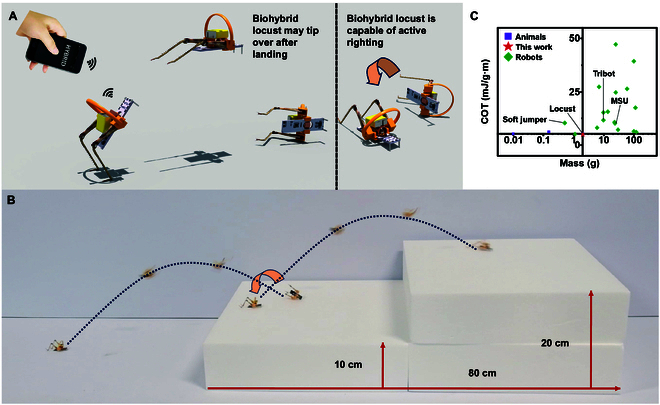
High-performance jumping microrobot. (A) Self-righting process of the biohybrid locust. (B) Biohybrid locust climbing stairs. (C) COT and mass of the biohybrid locust relative to existing jumping robots and insects.

The biohybrid locust achieves successive jumping through its repeatable jumping ability and self-righting capability, despite its small body size. High-performance jumping robots typically have unstable landings whereas locusts use their legs to right themselves quickly. As shown in Fig. 6A, the biohybrid locust utilizes a similar mechanism to right itself. Upon landing, the biohybrid locust uses a support ring to avoid getting stuck. Because it is initially in a tilted state, the periodic angular momentum generated when the biohybrid muscle actuators swing breaks the balance and causes the biohybrid locust to tilt in a specific direction and thus right itself, righting the robot (Movie S6). Figure 6B shows the biohybrid locust performing successive jumps to climb stairs (Movie S7), which highlights the synergistic maneuvering of its biohybrid muscle actuators and artificial joint for enhanced mobility and expanded applicability to obstacle-laden environments. As shown in Fig. 6C, the biohybrid locust boasts a smaller and lighter frame than existing jumping robots. The cost of transport (COT) is an important indicator of the energy efficiency of a robot or organism. A lower COT indicates that less energy is consumed per unit distance and thus a higher energy efficiency [19]. Figure 6D shows that the biohybrid locust has a substantially lower COT for long-distance jumping than existing small robots [19–21,23–37] and is comparable to the COTs of insects [38–40] (Table S1).

## Discussion

In this study, a sustainable materials-driven approach was utilized to repurpose discarded locust hindlegs as high-performance actuators in a jumping microrobot. The developed biohybrid locust achieved a remarkable jumping performance (600 mm distance, 150 mm height) with an ultralow power consumption (0.03 mW) while demonstrating steering and self-righting capabilities. Although the actuators exhibit a limited active duration of ~4 h, their hybrid architecture fundamentally addresses the critical challenges of insufficient explosive power and high energy consumption in conventional actuators. The developed prototype provides a novel paradigm for developing agile microrobots capable of operating in confined spaces with potential applications ranging from search-and-rescue missions to environmental exploration. Future efforts to standardize muscle tissue performance through bioengineering and to prolong operational stability may facilitate its practical deployment in dynamic real-world scenarios.

Although the biohybrid locust has impressive jumping capabilities, it still lacks the crawling ability of real locusts. Even though its jumps can be steered by asynchronous kicks, achieving a large steering angle remains a challenge. In addition, while it was able to climb stairs, it required manual assistance because the landing direction was random. The core objective of this study was to showcase the explosive force and jumping capabilities of the biohybrid muscle actuator in terms of the jumping height and distance. Thus, an active posture adjustment mechanism was not integrated. Locusts jumping in nature also have random landing directions, and they must first self-correct their posture before using their front and middle legs to adjust their direction. In the future, actively controllable dual-wheel structures [41] or steering mechanisms [36] will be introduced to enable directional adjustment on the ground. Endowing jumping microrobots with locust-like flexible crawling abilities should result in great advances in robotic motion control and task execution.

However, individual differences and growth processes of the locust hindlegs impacted the movement stability and consistency of the biohybrid muscle actuators. Breeding customized actuators through bioengineering technology will result in robots that can perform more stably and efficiently in complex environments. Although substantial challenges remain in functional maintenance, recent advances in tissue–material hybrid systems [10,42,43] have demonstrated the feasibility of combining ex vivo muscle tissues with artificial structures to achieve compact, responsive, and efficient actuation under electrical stimulation [14]. While current systems still largely depend on liquid environments for tissue viability, emerging encapsulation strategies [13] show potential for extending functional duration and improving mechanical robustness. We believe that continued progress in bio-hybrid actuation technologies will eventually enable standardized, high-performance “bio-artificial actuators” with tailored mechanical properties and extended operational lifespans. These research directions not only represent the cutting edge of future technology but also hold the potential to revolutionize the field of intelligent robotics and pave the way for a new era of flexible, precise, and efficient biomechanical systems.

## Materials and Methods

### Material acquisition

Adult female locusts (*Locusta migratoria*) with a length of 39.7 ± 8 mm and a mass of 2.0 ± 0.4 g were sourced from a breeding plant in Maoming City, Guangdong Province, China. A small group of 20 locusts was kept in an 80 cm × 80 cm cage and fed with fresh grass. The laboratory was kept at a temperature of 25 °C and a relative humidity of 60%. Before experiments, several locusts were gently stressed in a separate container, and some voluntarily severed their hindlegs. The discarded hindlegs were then collected for use. Locusts that had lost their hindlegs were collected in another box. The use of this animal was approved by the Animal Ethical and Welfare Committee (IACUC-2020026).

### Preparation of the biohybrid muscle actuators

Silver electrodes (785500, A-M Systems) were used for electrical stimulation of the locust hindlegs. These electrodes featured a polytetrafluoroethylene insulating layer with an inner diameter of 76.2 μm and an outer diameter of 139.7 μm. The implantation depth was controlled to 1 mm, and the implantation points were set differently based on the anatomical locations of the muscles. Flexor electrodes were placed 10 to 15 mm from the femorotibial joint, extensor electrodes were placed 3 mm from the proximal end of the femur, and the distance between the 2 electrodes was 1 mm. The electrodes were fixed by using a biological adhesive (4011, Loctite).

### Electrical stimulation protocols

A dual-channel electrical stimulation system was used to drive the biohybrid muscle actuator. The stimulation parameters were determined through systematic preliminary experiments and with reference to previous studies [7,16,22,44]. For the flexor muscles, square wave stimulation with a voltage of 2 V, a pulse width of 2 ms, and a frequency of 100 Hz was used. For the extensor muscles, the same voltage and pulse width were used, but the frequency was adjusted to 80 Hz based on their distinct frequency-response characteristics, as detailed in Results (Fig. 1). For the co-contraction process, the flexor muscles were first stimulated individually to retract the tibia. Then, co-stimulation of the flexor and extensor muscles was used to store energy by bending the SLP. Finally, the stimulation of the flexor muscles was stopped, which allowed the extensor muscles to act alone and achieve an explosive kicking motion.

### Microscopic imaging

A NIR-I fluorescence microscope (MCR2, Artemis intelligent imaging) was used to take images from a vertical angle. An 808-nm laser light source (LED-D1-805, Obeabt) was used for irradiation while the laser power was adjusted by using a laser controller (LEDOTB-1000, Obeabt). The locust hindlegs were attached to a transparent plastic culture dish with transparent tape for observation and experimentation. The laser light source was placed under the locust hindlegs, which were stimulated by a signal generator (33500B, Keysight) to observe the movements of the extensor and flexor muscles and joints. The image sequences were then made into movies (e.g., Movie S1).

### Force measurement

The force generated by driving the tibia from muscle contraction was measured to indirectly assess muscle contraction. In its natural state, the femur–tibia joint of the isolated locust hindleg had an angle of 80°. The femur was fixed to the experimental bench surface with the tibia oriented upward. A transducer (K3D40, ME) with a range of 2 N was mounted on the floor at 80° (Fig. S14A) to ensure that its loading direction was perpendicular to the tibia. The tibia was firmly connected to the loaded end of the transducer to prevent any rotation during muscle contraction. Thus, the transducer measured the isometric contraction force of the muscle. The flexor and extensor muscles were electrically stimulated by using 2 single pulse signals (Fig. S14B).

### Motion analysis

A high-speed camera (i-SPEED 221, iX Cameras) operating at 2,000 Hz was used to capture the kicking motion from a vertical perspective. The camera was mounted 500 mm above the isolated locust hindleg with the lens parallel to the horizontal plane. To track the movement, 3 small markers ~1 mm in diameter were placed at the trochanter–femur joint, femur–tibia joint, and tip of the tibia (Movie S2). The angle of the femur–tibia joint was calculated by connecting the 3 markers. The video was imported into motion analysis software (ProAnalyst, Xcitex) to calculate changes in this angle. The kicking process was normalized for all samples. The stopping of the electrical stimulation of the co-contraction process was regarded as the beginning of the kicking process, and the first rebound to zero velocity after the maximum kicking angle was reached was regarded as the end of the kicking process.

### Robot construction

The biohybrid locust was a board-mounted robot. As shown in Fig. 3A, the robot mainly comprised a support ring, battery, PCB, coil, 2 forelegs, a magnet, and 2 biohybrid muscle actuators. The PCB (30 mm × 8 mm × 1 mm) served as the robot platform and integrated the main control chip (TI CC2650), motor drive module (TI DRV8837), and power supply module (Fig. S8). Two channels of the main control chip controlled the digital output of the motor drive module, which in turn drove the artificial rotary joint. For each biohybrid muscle actuator, 2 channels of the main control chip drove the flexor muscle and 1 channel drove the extensor muscle, as illustrated in Fig. 3B. The parts were glued together (PR100, 3M). A 25-mAh lithium-ion battery was used to power the biohybrid locust. The main control chip was connected to a mobile phone via Bluetooth. The biohybrid locust had a total mass of 2 g.

### Jump experiments

A mobile phone was utilized to send commands to the biohybrid locust, which performed the corresponding actions. Two 3-axis transducers (K3D40, ME) were used to record the bouncing force of the robot during jumping at a sampling frequency of 2,000 Hz. Two high-speed cameras (i-SPEED 221, iX Cameras) operating at 2,000 Hz recorded the turning process of the robot during jumping. A 3-dimensional motion capture system (V5, Vicon) equipped with 10 infrared cameras with a frequency of 400 Hz was used to track the jumping trajectory, as illustrated in Fig. 5A. The biohybrid locust was placed in the same position and orientation for each jump.

## Data Availability

All data relevant to this study are available in the manuscript and the Supplementary Materials. Raw data are available from the corresponding authors upon reasonable request.

## Supplementary Materials

Supplementary 1 Notes S1 to S8 Figs. S1 to S14 Tables S1 to S4 Movies S1 to S7
