# Supplementary material for: Locust-Derived Biohybrid Muscle Actuators for Low-Power Explosive Jumping
Source: Research (Wash D C). 2025 Oct 21;8:0943. doi: 10.34133/research.0943 (PMC12538119; doi:10.34133/research.0943)
Supplement: Supplementary 1 — Notes S1 to S8 Figs. S1 to S14 Tables S1 to S4 Movies S1 to S7 [file research.0943.f1.zip › Supplementary_Materials.docx]

Supplemental Materials

Title

Locust-Derived Biohybrid Muscle Actuators for Low-Power Explosive Jumping

**Authors**

Peng Liu^1^†, Yao Li^1,2,3,4^†^*^, Songsong Ma^5*^, Yunhao Si^1^, and Bing Li^1,2,3,4*^

**Affiliations**

^1^School of Robotics and Advanced Manufacture, Harbin Institute of Technology, Shenzhen, Shenzhen, 518055, China

^2^State Key Laboratory of Robotics and Systems, Harbin Institute of Technology, Harbin, China

^3^Guangdong Provincial Key Laboratory of Intelligent Morphing Mechanisms and Adaptive Robots

^4^Key University Laboratory of Mechanism & Machine Theory and Intelligent Unmanned Systems of Guangdong

^5^School of Mechanical and Electrical Engineering, the Shenzhen Polytechnic University, Shenzhen, 518055, China.

†These authors contributed equally

^*^Correspondence: liyao2018@hit.edu.cn; masongsong@szpu.edu.cn; libing.sgs@hit.edu.cn

**Note S1. The muscles of the hind legs of locusts.**

The locust hindleg muscles are mainly located on the femur. The extensor is large and occupy the entire upper region of the femur. When the extensor contracts, the tibia opens outward. The flexors are small and located on the lower side of the femur, and when the flexors contract, the tibia tucks inward. The extensors and flexors form a pair of antagonistic muscles that control the rotation of the tibia around the center of rotation[1]. Flexors are controlled by 11 nerve[2], including 9 excitatory and 2 inhibitory nerves. Inhibitory nerve signals act at the end of the co-contraction storage phase to stop the contraction or trigger a kicking or jumping action. The flexors contract rapidly, reaching maximum contractile force within 100 ms after receiving excitatory nerve signals; the extensors contract slowly, reaching maximum contractile force within 150 - 350 ms after receiving excitatory nerve signals.

**Note S2. Electrode implantation.**

Electrical signal was achieved by electrodes implanted on the surface of the isolated locust hindleg muscle to control muscle contraction. It was found that stable muscular contraction was achieved in both medial and lateral directions of the hindleg. As shown in Fig. S1A, when the electrodes were implanted from the lateral side of the hindleg, the flexor implantation position was 10 mm away from the FT joint (Femur-tibia joint); As shown in Fig. S1B, when the electrodes were implanted from the medial side of the hindleg, the flexor implantation position was 15 mm away from the FT joint. In order to minimize the effect of the extensor stimulation on the flexor stimulation, the extensor implantation position was 3 mm away from the proximal end of the femur regardless of whether the implantation position was from the medial side or lateral side, and the implantation point of electrode pairs differed in distance by 1 mm. Metal-silver electrodes (785500, A-M Systems) featuring an inner diameter of 76.2 μm and an outer diameter of 139.7 μm with a Teflon insulating layer were used. Prior to implantation, the electrode tip was burnt into a small ball of ~ 1 mm in diameter. The electrode was implanted to a depth of ~ 1 mm and the electrode was secured with bio-adhesive (4011, Loctite).

**Note S3.** **Modelling the response of the flexor and extensor of locust hindlegs.**

Firstly, the single impulse response of the muscle was modelled. Since the signal duration was short compared to muscle response, it's approximated as a shock response. Assuming that the input and output relationship of the process was linear, time-domain impulse responses were converted to the frequency domain using the *Fourier Transform* and the data were fitted using the following model:

$$G(s)=\frac{N(s)}{D(s)}=\frac{b_{m}s^{m}+b_{m-1}s^{m-1}+\cdots+b_{0}}{s^{n}+a_{n-1}s^{n-1}+\cdots+a_{0}}$$

Where *m* - the number of transfer function zeros, *n* - the number of transfer function poles, the coefficients *b_m_*, *b_m-1_* ··· *b_0_*, *a_n-1_*, *a_n-2_*···*a_0_* were estimated using least squares:

$$J=\sum_{k=1}^{r} \left|G(j\omega_{k})D(j\omega_{k})-N(j\omega_{k})\right|^{2}$$

$$\left[a_{n-1}\text{ }a_{n-2}\text{ }\cdots\text{ }a_{1}\text{ }a_{0}\text{ }b_{m}\text{ }b_{m-1}\text{ }\cdots\text{ }b_{1}\text{ }b_{0}\right]^{T}=(\mathbf{X}^{T}\mathbf{X})^{-1}\mathbf{X}^{T}\mathbf{y}$$

Where:

$\mathbf{X}=\left[\begin{matrix} G(j\omega_{1})(j\omega_{1})^{n-1} & \cdot\cdot\cdot& G(j\omega_{1})(j\omega_{1}) & G(j\omega_{1}) & -(j\omega_{1})^{m} & \cdot\cdot\cdot& -(j\omega_{1}) & -1 \\ G(j\omega_{2})(j\omega_{2})^{n-1} & \cdot\cdot\cdot& G(j\omega_{2})(j\omega_{2}) & G(j\omega_{2}) & -(j\omega_{2})^{m} & \cdot\cdot\cdot& -(j\omega_{2}) & -1 \\ \cdot& \cdot& \cdot& \cdot& \cdot& \cdot& \cdot& \cdot\\ \cdot& \cdot& \cdot& \cdot& \cdot& \cdot& \cdot& \cdot\\ \cdot& \cdot& \cdot& \cdot& \cdot& \cdot& \cdot& \cdot\\ G(j\omega_{r})(j\omega_{r})^{n-1} & \cdot\cdot\cdot& G(j\omega_{r})(j\omega_{r}) & G(j\omega_{r}) & -(j\omega_{r})^{m} & \cdot\cdot\cdot& -(j\omega_{r}) & -1 \end{matrix}\right]$ $\mathbf{y}=\left[\begin{matrix} -G(j\omega_{1})(j\omega_{1})^{n} & -G(j\omega_{2})(j\omega_{2})^{n} & \cdots& -G(j\omega_{r})(j\omega_{r})^{n} \end{matrix}\right]$

The number of poles and zeros in the muscle model affects fit. High-order models fit better but may overfit. Muscle impulse response was assessed by: PF (Peak Force), the maximum value of the recorded force; FTI (Force Time Integral), the area under the force trajectory; HRT (Half Relaxation Time), the time required for 90% of the maximum force to drop to 50%; LRT (Late Relaxation Time), the time required for 40% to drop to 10%; and TPT (Time to Peak Tension), the time from zero to 90%. The poles and zeros of the model were gradually increased from small to large, and the muscle model was finalized based on the degree of fit. The isotonic contraction was modelled using continuous impulses, applied to the single impulse response model, and compared with the experimental data.

The second-order and third-order models for the flexor were calculated, respectively:

$$TF_{2}=\frac{951.5}{s^{2}+127.5s+7639}$$

$$TF_{3}=\frac{\text{ 6.886}e04}{s^{3}+257.7s^{2}+3.935e04s+1.786e06}$$

The average force curve of flexor and the fitting curve of the model were plotted as shown in Fig. S2, and the fitting degree of the second-order model was 82.76%, with an obvious fluctuation at the beginning of the curve; the fitting degree of the third-order model was 95.94%. The evaluation parameters of the two models for flexor were listed and compared with the experimental data as shown in Table S2. The four parameters of PF, FTI, HRT and LRT of the third-order model were closer to the values of the average curve, and there was no significant difference between the second-order model and the third-order model for the TPT parameter, so this third-order model was chosen as the mathematical model of the flexor impact response.

The two fifth-order models for the extensor were calculated:

$$TF_{5}=\frac{\text{ 2.187}e10}{s^{5}+877.5s^{4}+6.108e05s^{3}+3.658e07s^{2}+1.397e09s+1.843e10}$$

$$TF'_{5}=\frac{6.22e04s^{2}+2.548e05s+4.058e08}{s^{5}+99.61s^{4}+1.244e04s^{3}+6.805e05s^{2}+2.466\times10^{7}s+3.421e08}$$

The mean force curves of the extensor and the fitting curves of the models were plotted as shown in Fig. S3, and the fitting degree of the first fifth-order model was 90.26 %, and the fitting degree of the second fifth-order model was 95.04 %. The evaluation parameters of the two models for extensor were listed and compared with the experimental data as shown in Table S3. The fifth-order model 2 has more evaluation parameters closer to the average curve than the fifth-order model 1, so this fifth-order model 2 was chosen as the mathematical model of the extensor.

A continuous pulse signal with a duration of 400 ms and parameters of 2 V-2 ms-30 Hz and 2 V-2 ms-90 Hz was applied to the flexor and flexor mathematical model, respectively. When the frequency of the continuous waveform was set to 30 Hz, both experimental and simulated data showed obvious fluctuation phenomena along with the pulse of the electrical stimulation waveform, which is due to the fact that the frequency of the electrical stimulation waveform was too low, and flexor was not affected by the previous pulse until the previous pulse was contraction induced by the previous pulse is released before it is affected by the next pulse. In order to achieve full contraction of the flexor under a single pulse, a new mathematical model of isotonic contraction of the flexor was re-established by setting the parameter of a single pulse to 5 V-3 ms, referring to the study of Wilson et al. [3], as follows:

$$TF_{3}=\frac{7.787e04}{s^{3}+273.6s^{2}+2.499e04s+8.046e05}$$

A continuous electrical stimulation waveform of 400ms with the pulse signal parameter set to 5 V-3 ms and the frequency from 30 Hz to 90 Hz was applied to the locust hindleg flexor and the improved mathematical model, and the isotonic curve of the flexor was obtained as shown in Fig. S4. When the frequency of electrical stimulation was set from 30Hz to 90Hz, the maximum flexor force obtained from the experimental data and the simulated data were the same, for example, at 30Hz, the maximum flexor force produced was both 0.011 N, and at 90 Hz, the maximum flexor force produced was both 0.035 N. However, there is still a difference between the simulated data and the experimental data, in the process of force gradually increasing from 0 to the maximum value, the simulated data has a faster speed. This phenomenon is most obvious in the frequency of 80Hz. The experimental data need 0.2 s to reach the maximum value, while the simulated data has reached the maximum value of the flexor force in 0.07 s. For flexor electrical stimulation, this mathematical model based on a single pulse does not take into account the fact that the cumulative effect of flexor force under multiple pulses is affected by other factors, but rather it is more desirable to simply add up the forces generated by individual pulses.

For the extensor, when the parameters of the continuous 400 ms electrical stimulation pulse were set to 2 V-2 m-30 Hz and 2 V-2 ms-100 Hz, the obtained isotonic curves of the extensor are shown in Fig. S5. When the frequency of electrical stimulation was 30Hz, there was no obvious fluctuation in both experimental and simulated data, which indicated that the extensor muscle had contracted sufficiently under the current electrical stimulation parameter and was close to the state of tonic contraction, and the maximal force in both experimental and simulated states was around 0.14 N. However, the simulated data reached the maximal value of the force at 0.1 s, whereas the experimental data was always in the ascending stage. When the frequency of electrical stimulation was set to 100 Hz, the simulated values of extensor force were much larger than the experimental measurements, and the simulated data also obtained the maximum value of force in a shorter period, while the experimental measurements were still in the state of rising all the time, and levelled off at 0.4 s. The simulated values of extensor force were much larger than the experimental measurements when the frequency of electrical stimulation was set to 100 Hz.

The structural characteristics of the extensor itself dictate that the mathematical model of the muscle under continuous electrical stimulation pulses cannot be a simple linear summation of the mathematical model of the muscle under a single pulse waveform. The complexity of force generation in an irregular muscle distribution such as the extensor cannot be described using only one mathematical model for all electrical stimulation parameters. In the study by Wilson et al. a second-order model was used for fitting when the frequency was low and the number of pulses was small; a linear third-order model was used for fitting when the frequency was moderate and the duration of electrical stimulation was long; and a generalized model was proposed when the input was high frequency and a large number of pulses, which was a second-order model and contained two nonlinear terms[4]. However, this generalized model parameterizes the calcium ion concentration into account, which would largely limit the use of the locust hindleg as a biological actuator if the muscle modeled were closely related to calcium ion concentration.

Since the isometric force of the extensor cannot be represented by a uniform mathematical model, the isometric force of the extensor at different electrical stimulation frequencies was measured experimentally. As shown in Fig. 2C of the manuscript, when the electrical stimulation frequency was less than 60 Hz, the isometric force of the extensor increased with the increase of the frequency, and the isometric force of the extensor did not continue to increase when the electrical stimulation frequency is greater than 60 Hz. According to practical needs, the force representation could be obtained by extensor force curve fitting based on the experimental data. The extensor force was modeled as follows, achieving a high level of accuracy within the first 400 ms:

$$F(t)=\frac{p_{1}t^{4}+p_{2}t^{3}+p_{3}t^{2}+p_{4}t+p_{5}}{t^{3}+q_{1}t^{2}+q_{2}t+q_{3}}$$

The modeling parameters are provided in Table S4.

The extensor is fully contracted at high frequencies (more than 70 Hz), while the extensor force is modulated by adjusting the stimulus duration at a constant frequency, so the isotonic contraction of the extensor in full contraction under continuous stimulation was modelled:

$$TF_{4}=\frac{-0.001s^{4}+0.1347s^{3}-1.1534s^{2}+5.0304s-9.792}{0.0541s^{4}+0.0509s^{3}-1.5548s^{2}+6s}$$

**Note S4. Determination of the bio-actuator’s rebound characteristics.**

The femur was fixed by the beeswax to a horizontal tabletop, and the tibia was placed facing upward. The weight loading of the tibia tip was realized by pasting a beeswax block with the following loading masses: 0 mg, 20 mg, 40 mg, 60 mg, 80 mg, and 100 mg, as shown in Fig. S6A. A high-speed camera (i-SPEED 221, iX Cameras) was used to take pictures from a direction perpendicular to the joint plane, and the measured joint angle *θ* was derived in motion analysis software (ProAnalyst, Xcitex). When measuring the rebound moment of tibia abduction, the femur was fixed in an 80 deg inclined position to the horizontal plane, and the rest of the treatment was consistent with the measurement of the rebound moment of tibia internal retraction, as shown in Fig. S6C. Each loading of the tibia tip was in dynamic static equilibrium. The force analysis of the loading process is shown in Fig. S6B&D, where *m_1_* is the loaded mass, *m_0_* is the tibial mass which is 0.014 g, *l* is the tibial length which is 20 mm. Multiple samples were chosen for this experiment to reduce the effect of sample differences.

As the load weight increases or decreases, the joint angle gradually decreases/increases, which can be approximated as a linear change. When measuring the internal rebound torque:

$$M=m_{1}gl\cos\theta+\frac{1}{2}m_{0}gl\cos\theta$$

$$\theta=69.59-0.31m_{1}$$

When measuring the external rebound torque:

$$M=m_{1}gl\sin(10+\theta)+\frac{1}{2}m_{0}gl\sin(10+\theta)$$

$$\theta=67.38+0.63m_{1}$$

**Note S5. Evaluation of energy consumption in bio-actuators.**

The muscle tissue between the electrode pairs can be equated to a circuit with a resistor and capacitor in series and then in series with the resistor, as shown in Fig. S8A. Unidirectional pulses were applied to the flexor and extensor of the bio-actuator and the changes in current were recorded with an oscilloscope (DSOX1204G, Keysight). The power consumed by the electrical pulses to stimulate the muscles during a single kick was calculated according to the formula for work done. A thermal camera (TV40, Fluke) captured temperature changes on the surface of the bio-actuator from a vertical angle. The camera was mounted 500 mm above the isolated hind leg with the lens parallel to the horizontal plane. A kick control signal was applied to the bio-actuator every 1 second.

The input power for a pair of hindleg actuators was ~0.03 mW, as shown in Fig. S8B, which is markedly lower than the power required for artificial miniature robots (ranging from 100-1000 mW)[5]. This significant difference is attributed to the fact that the kinetic energy of the bio-actuator originates from its internal mitochondria, rather than relying on electrical control signals. Despite the low input power, the biological energy consumed by leg kicking is still considerable. A ~0.3 ℃ temperature rise was observed after a leg kick, implying a large portion of the biological energy was dissipated as heat, as shown in Fig. S8C&D. Due to the lack of fluid replenishment, the continuous consumption of internal biological energy ultimately leads to the cessation of leg kicking.

**Note S6. Development of artificial rotary joint.**

The natural jumps of locusts require not only fast hindleg stretching, but also the auxiliary movements of other joints, e.g., trochanter. Specifically, the locust initially rotates its trochanter joint to elevate the entire leg, followed by retracting the tibia. Subsequently, the trochanter joint is pressed down for ground contact, and finally enabling a jump. Hence, our biohybrid locust needs to incorporate an artificial rotary joint.

The joint, comprising a support ring, coil, and magnet (as illustrated in Fig. S9), rotates in response to variations in the magnetic field generated by the coil. By altering the current direction, as shown in Fig. S10A, the direction of joint rotation can be reversed, enabling leg lifting (with a 30-degree upward rotation) and pressing (with a 10-degree downward rotation). The support ring is a ring-shaped holder fabricated by 3D printing. Its upper part serves as a ring for passive support, while the lower part consists of two slots for equipping bio-actuators and a cantilever for placing the magnet, as illustrated in Fig S9. As shown in Fig. 3A of manuscript, the shaft on the slots forms a rotating vice with the platform. The coil is secured to the fixed platform. The coil drives the support ring to rotate when energized.

The support ring serves as the robot's passive stabilizing structure, with its core functions including: (1) Limiting the range of tipping: preventing the robot from completely toppling over during vibrations, ensuring that the center of gravity remains within a recoverable range (similar to the base of a “self-righting” toy). (2) Providing a pivot point: forming an instantaneous rotational axis by contacting the ground, enabling angular momentum to be converted into an effective overturning torque (τ = r × F). (3) Energy direction: Utilizing the symmetry of the ring structure, vibration energy is directed toward the center of gravity displacement direction, aiding the realignment process. Video S6 clearly demonstrates how the robot gradually realigns itself through vibration after the support ring contacts the ground (through the synergistic action of angular momentum and the pivot point).

The output torque of the artificial rotary actuator was calculated using numerical simulation software (Maxwell 2021 R1, Ansys). The magnets were made of *N35 NdFeB* with a diameter of 2.5 mm and a length of 3 mm. *Br* (remanent magnetism) was 1.18 T, *Hcj* (intrinsic coercivity) was 960 kA/m, *Hcb* (coercivity) was 860 kA/m, and *(BH)_max_* (maximum magnetic energy product) was 275 ± 12 kJ/m^3^. The coils were made of coiled copper wire, with an inner diameter of 5.5 mm, an outer diameter of 6.5 mm, a length of 3.7 mm, and a wire gauge of 0.07 mm. The number of turns were set to 200 (40% slot fullness), 250 (50% slot fullness), 300 (60% slot fullness), 350 (70% slot fullness). The maximum actuator rotation was 55 degrees. A 3-axial gaussmeter (G93, Coliy) was utilized to measure the actual magnetic field generated by the coil and the derived data was compared with the simulated data. The coil was fixed, as shown in Fig. S11. The gaussmeter probe was fixed on a sliding table and moved along the guide rail. The copper mesh was shielded from the ambient magnetic field. Multiple measurements of the data were taken and averaged.

Fig. S10B presents the simulation results of magnetic torque, with numerical calculations aligning closely with the measured magnetic field strengths shown in Fig. S10C. This confirms the validity of our numerical calculation method. The simulation results revealed the torque generated by coil actuators with varying numbers of turns. To overcome the effects of gravity, the induced torque needs to be at least 27 μN·m. Thus, a 200-turn coil actuator cannot provide enough torque at a 30-deg rotation (only 24 μN·m), whereas a coil with 250 turns can generate sufficient torque (over 31 μN·m).

**Note S7. Integration of control boards with jumping robot systems.**

As shown in Fig. 1A, the robot mainly consisted of a support ring, a battery, a PCB (printed circuit board), a coil, two forelegs, a magnet, and two isolated locust hindlegs. The PCB, which served as the robot platform, was 30 mm long, 8 mm wide, and 1 mm thick. The PCB integrated the main control chip TI CC2650, the motor drive module TI DRV8837 and the power supply module, as shown in Fig. S12. Two channels of the TI CC2650 controlled the digital output of the TI DRV8837, which in turn drove the artificial rotary actuator. Its two channels drove the flexor of the left-leg actuator and one channel drove the extensor of the left-leg. For the right-leg actuator, the same applied, as illustrated in Fig. 1B. The parts were glued using glue (PR100, 3M). The battery was a 25 mAh lithium-ion battery. The TI CC2650 was connected to mobile phone via Bluetooth. The total mass of the robot is 2 g.

**Note S8. Modelling of steering jump dynamics.**

To gain a deeper understanding of its steering jumps, the steering jump dynamics of the biohybrid locust was modelled as illustrated in Fig. S13A. It was assumed that the positions of the components of the bio-hybrid jumping robot were fixed differently. The mass *m* was 2 g, with its center of gravity situated on the upper surface of the magnet. The inertia [*I_x_*, *I_y_*, *I_z_*] was [0.114 0.073 0.122] kg·mm^2^. The local coordinate system [*x*, *y*, *z*] aligned with the center of gravity. Before jumping, the initial direction of the left and right hindleg force forms *β* angle with the vertical direction, which is the initial angle of robots jumping. Under the influence of different forces on the left (*F_l_*) and right (*F_r_*) sides, the rigid body rotates in three dimensions with angular velocity *w_k_(t)* around *k(t)*, and the angle between *k(t)* and the *z-axis* varies over the time, denoted as *ω(t)*. The force arms of the bouncing force were *l_l_* and *l_r_*, the duration of the whole jump was *T*, and the calculated step time was *Δt*. The simulated jumping trajectory of the bio-hybrid jumping robot was solved by substituting the force profile recorded by the force sensor into the established dynamics model. The velocity, displacement, angular velocity, body rotation angle, and deflection angle during the jump can be calculated using the following equations:

$$v=\frac{\triangle t}{m}\left(F_{l}+F_{r}\right)$$

$$S=\int_{0}^{T} v dt$$

$$\omega=\frac{\triangle t}{I}(l_{l}\times F_{l}+l_{r}\times F_{r})$$

$$\alpha=\int_{0}^{T} \omega dt$$

The outputs are the jumping trajectories of the robot, including the velocity, rotational angular velocity, and deflection angle. With the recorded force sensor data, we substituted the force application curves into the dynamics model to simulate the jump trajectory of the biohybrid locust. The model's accuracy was validated by comparing the simulated and actual jumping trajectories, which differed by less than 2 cm, as shown in Fig. S13C. This close alignment strongly supports the validity of our kinetic model.


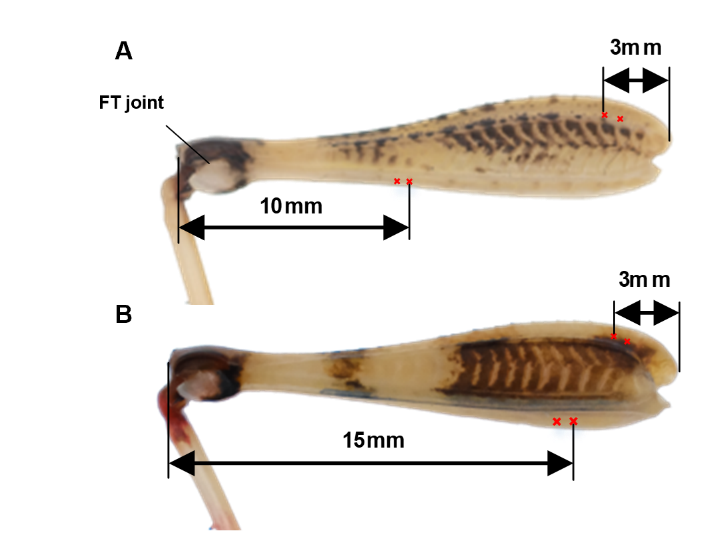


Fig. S1. Schematic diagram of electrode implantation positions. (A) The lateral electrode implantation position. (B) The medial electrode implantation position.


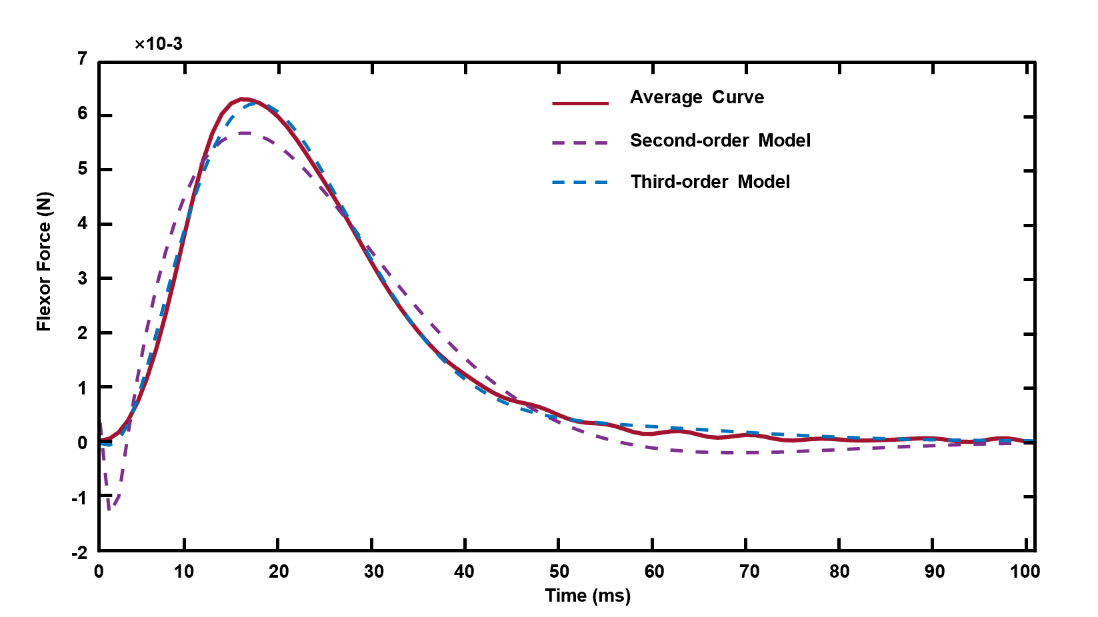


Fig. S2. Isotonic contraction force model of flexor.


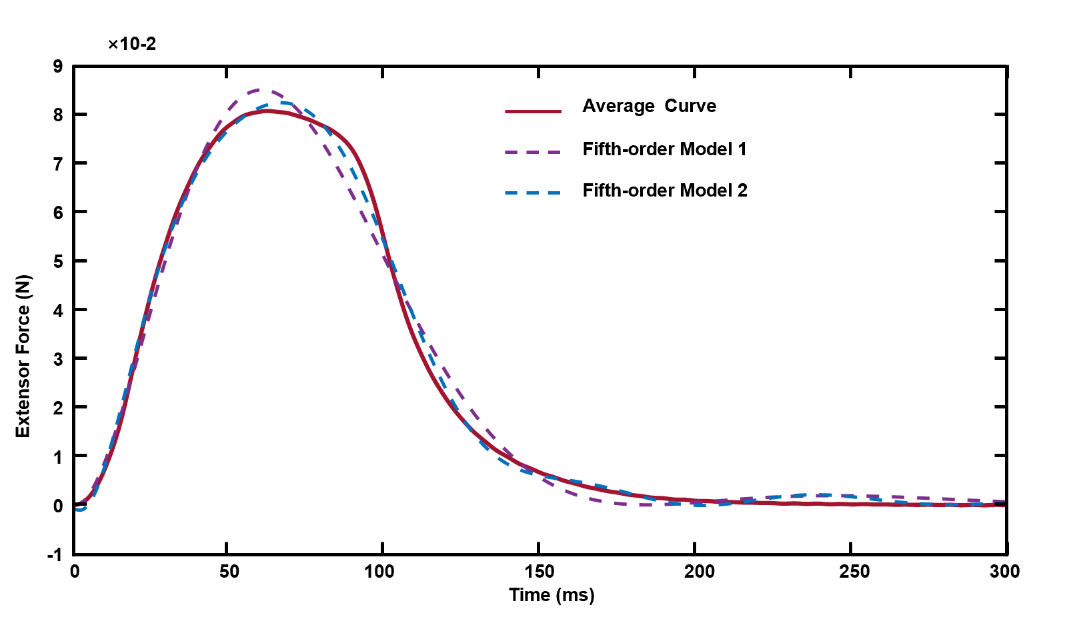


Fig. S3. Isotonic contraction force model of extensor.


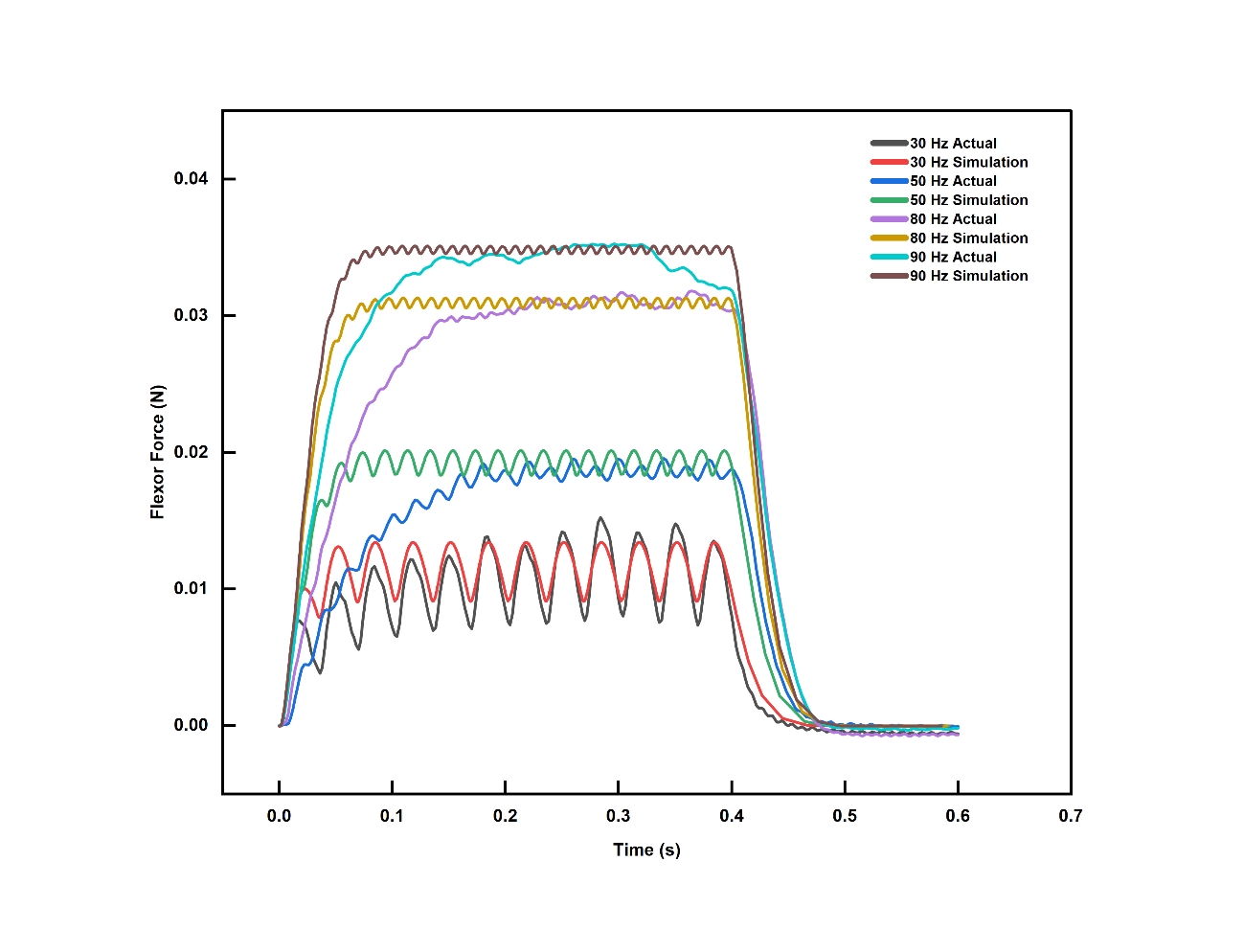


Fig. S4. Comparison curves of experimental data and simulated data of flexor under continuous pulse stimulation.


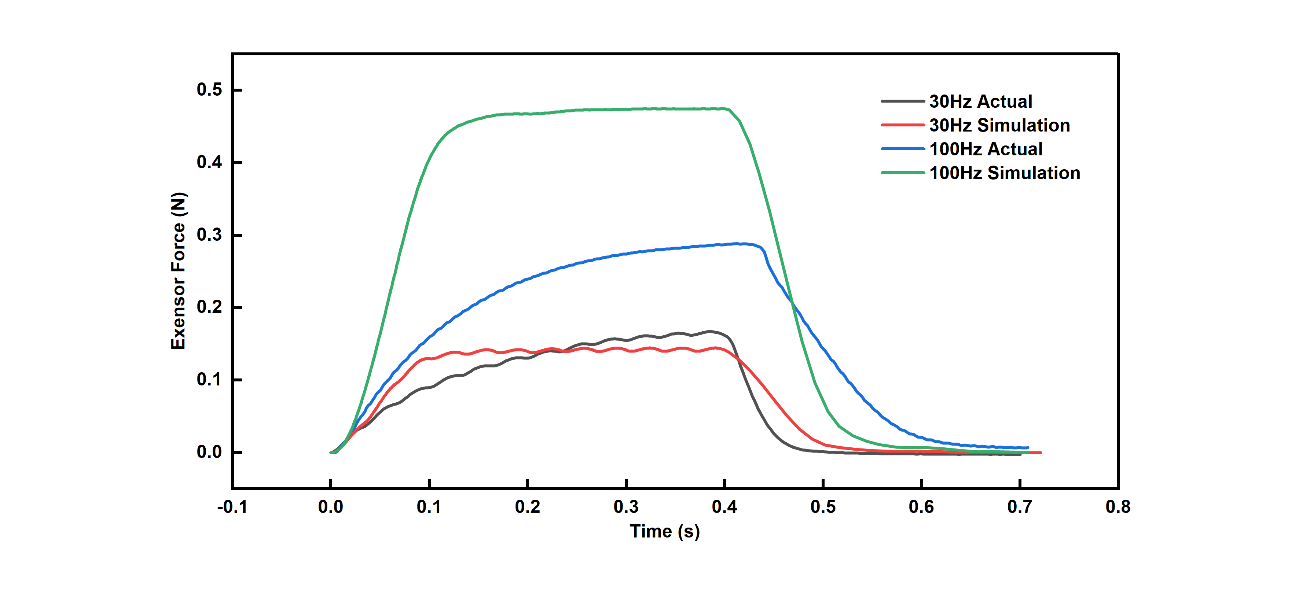


Fig. S5. Comparison of experimental data and simulated data of extensor under continuous pulse stimulation.


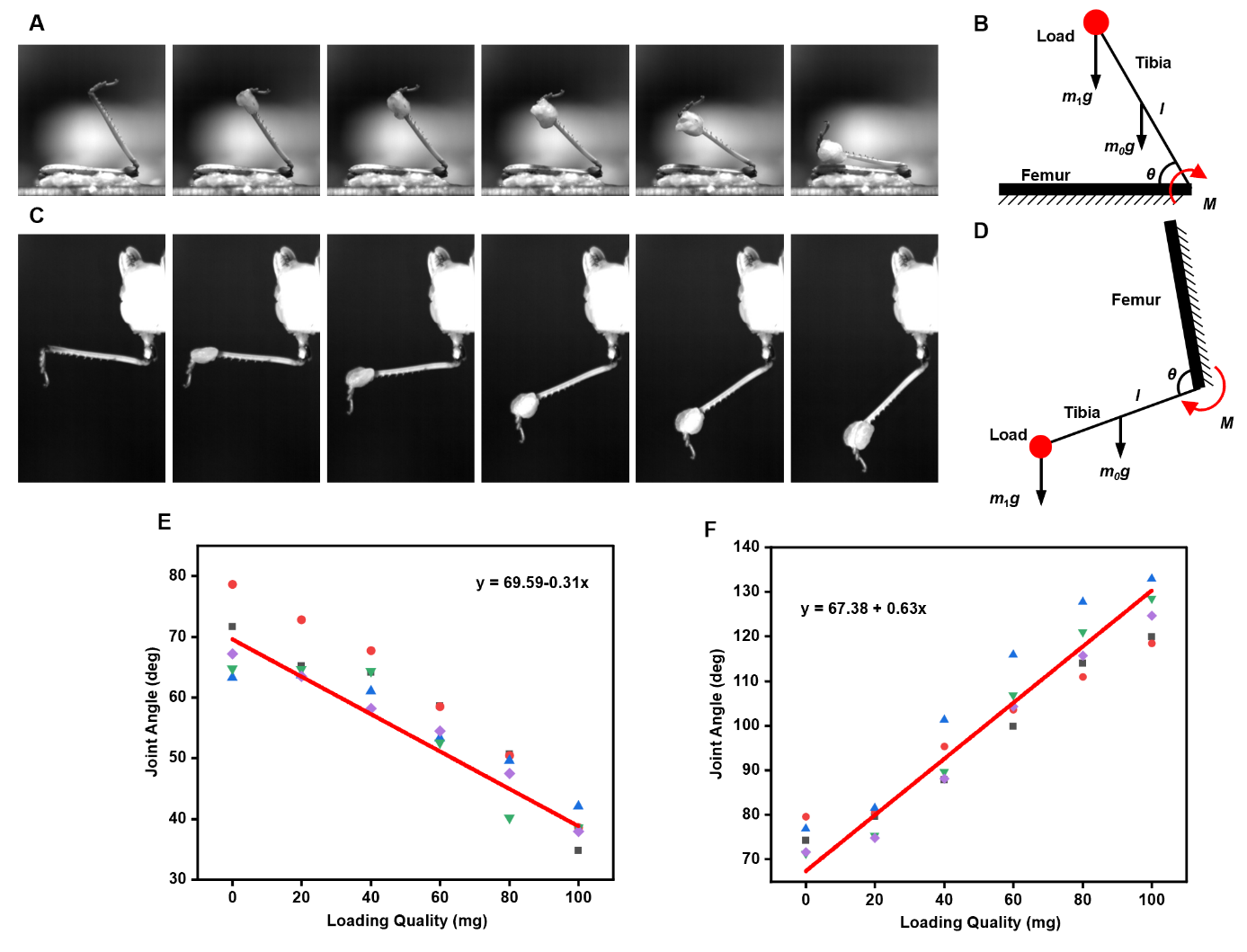
Fig. S6. Measurement and analysis of retraction torque in locust hindleg joints. (A) Procedure for tibial adduction torque measurement. (B) Mechanical modelling of the bio-actuator during the tibia adduction torque measurement. (C) Procedure for tibial abduction torque measurement. (D) Mechanical modelling of the bio-actuator during the tibia abduction torque measurement. (E) Relationship between loaded mass and joint angle during tibia adduction (N = 5 hindlegs, n = 5 trials). (F) Relationship between loaded mass and joint angle during tibia abduction (N = 5 hindlegs, n = 5 trials).


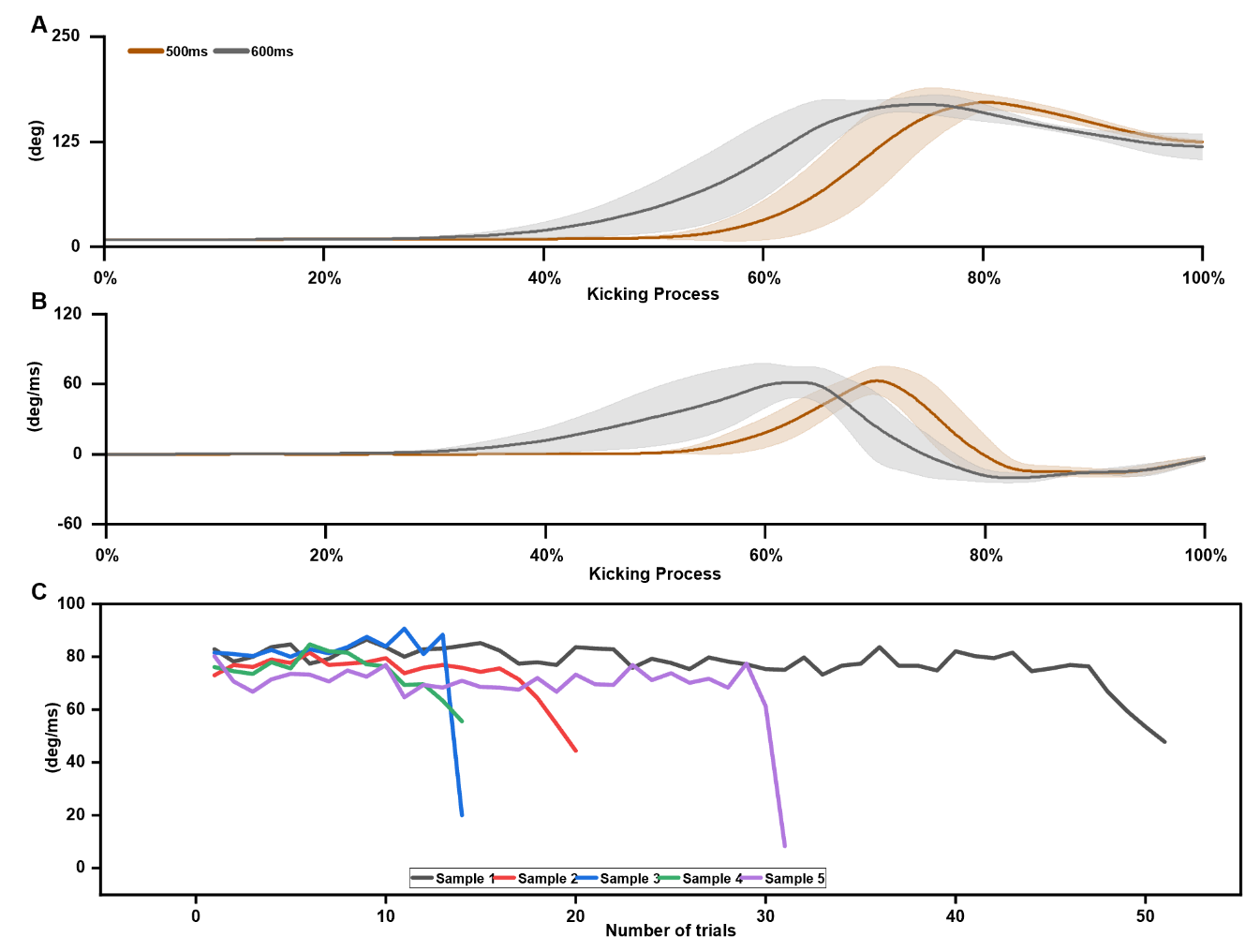


Fig. S7. Kicking characteristics of isolated locust hindlegs. (A) and (B) Kicking angle curves in (A) and angular velocity curves in (B) under co-contraction times of 500 ms and 600 ms (N = 16 hindlegs, n = 16 trials), where the solid line represents the mean and the shaded area represents the variance. (C) Decay curves of maximum kick velocity for multiple samples in a continuous kicking experimental setting. Different color curves represent different samples.


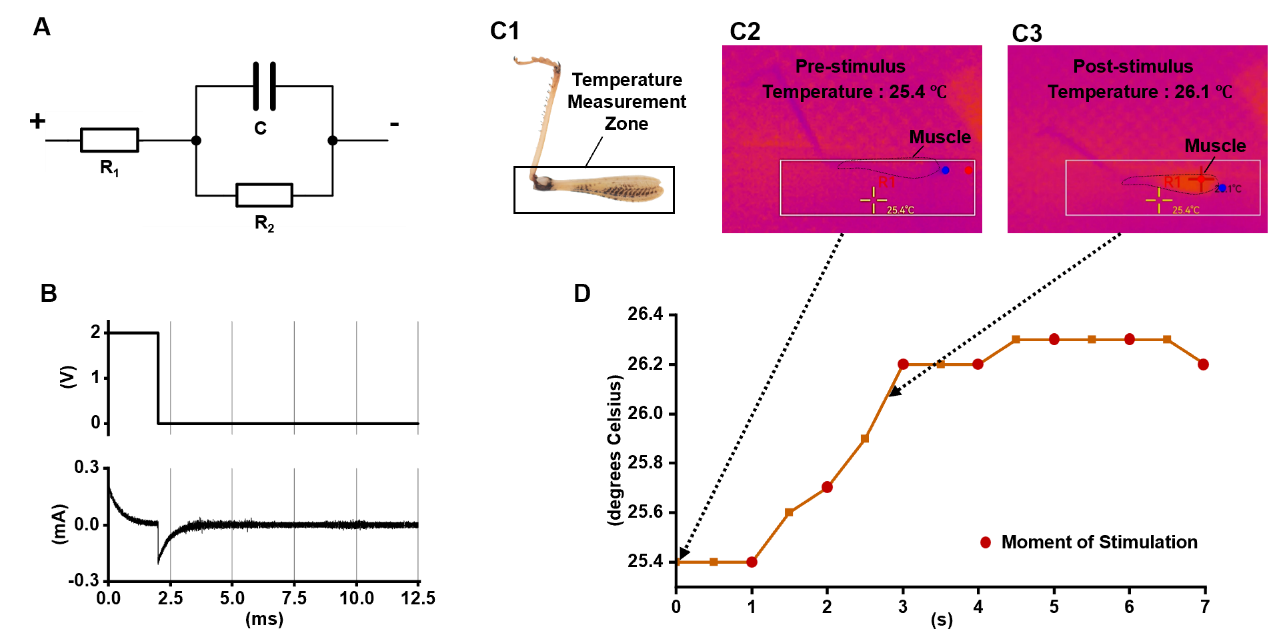


Fig. S8. Measurement and analysis of energy consumption characteristics of bio-actuators. (A) Equivalent circuit of muscular tissue. (B) Voltage signal applied to the muscle and current flowing through it. (C1), (C2) and (C3) Infrared footage of the kicking process. After several kicks, there was a significant temperature rise on the surface of the bio-actuator. (D) Temperature change of the surface of the bio-actuator over the time of the experiment.


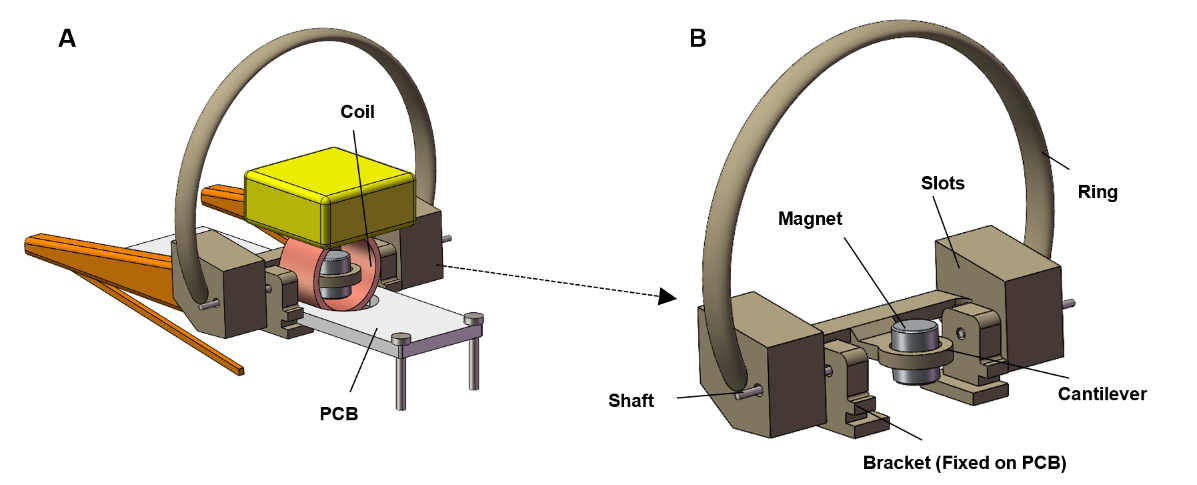


Fig. S9. Schematic representation of the 3D structure of an artificial rotary joint and its mounting position on the robot.


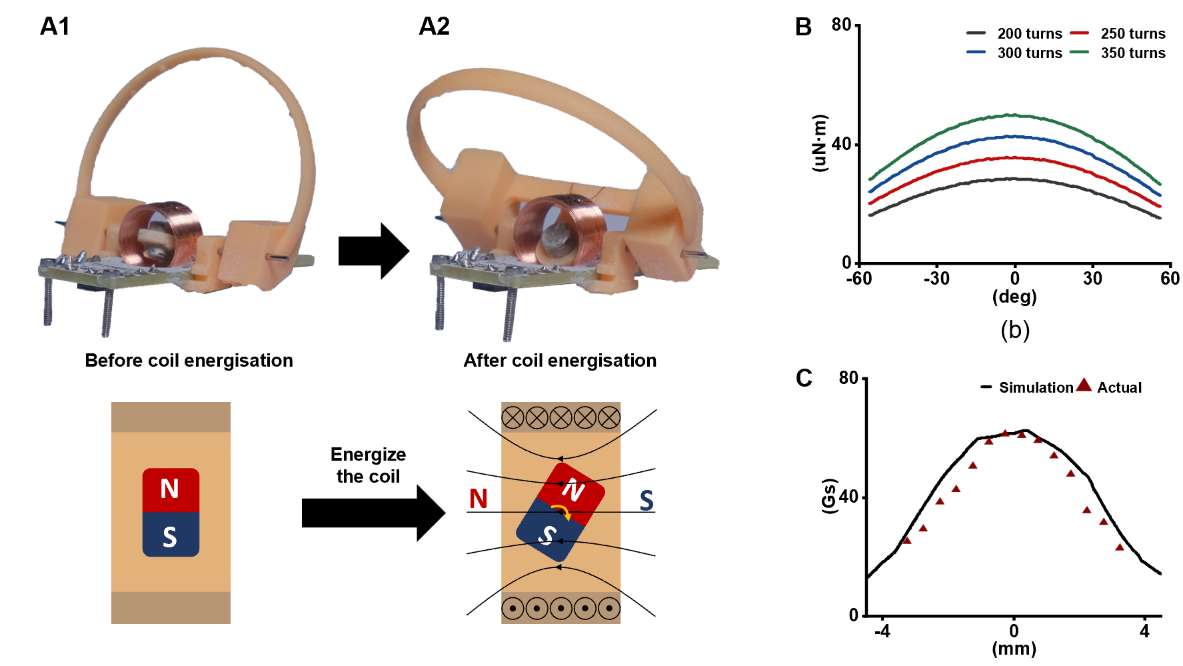


Fig. S10. Design and analysis of artificial rotary joint. (A1) and (A2) Schematic representation of the rotational principle of artificial joint. Changing the direction of current can change the rotation direction of the joint. The fork sign indicates the direction of current flow from outside the paper to inside the paper, the dot sign is the opposite, and the curves with arrows represent the magnetic inductance and its direction. Inside the coil, the lines of magnetic induction point from the S-pole to the N-pole. (B) Torque curves of the electromagnetic servo with different coil turns. (C) Magnetic fields analyzed through simulations were compared with actual measured values.


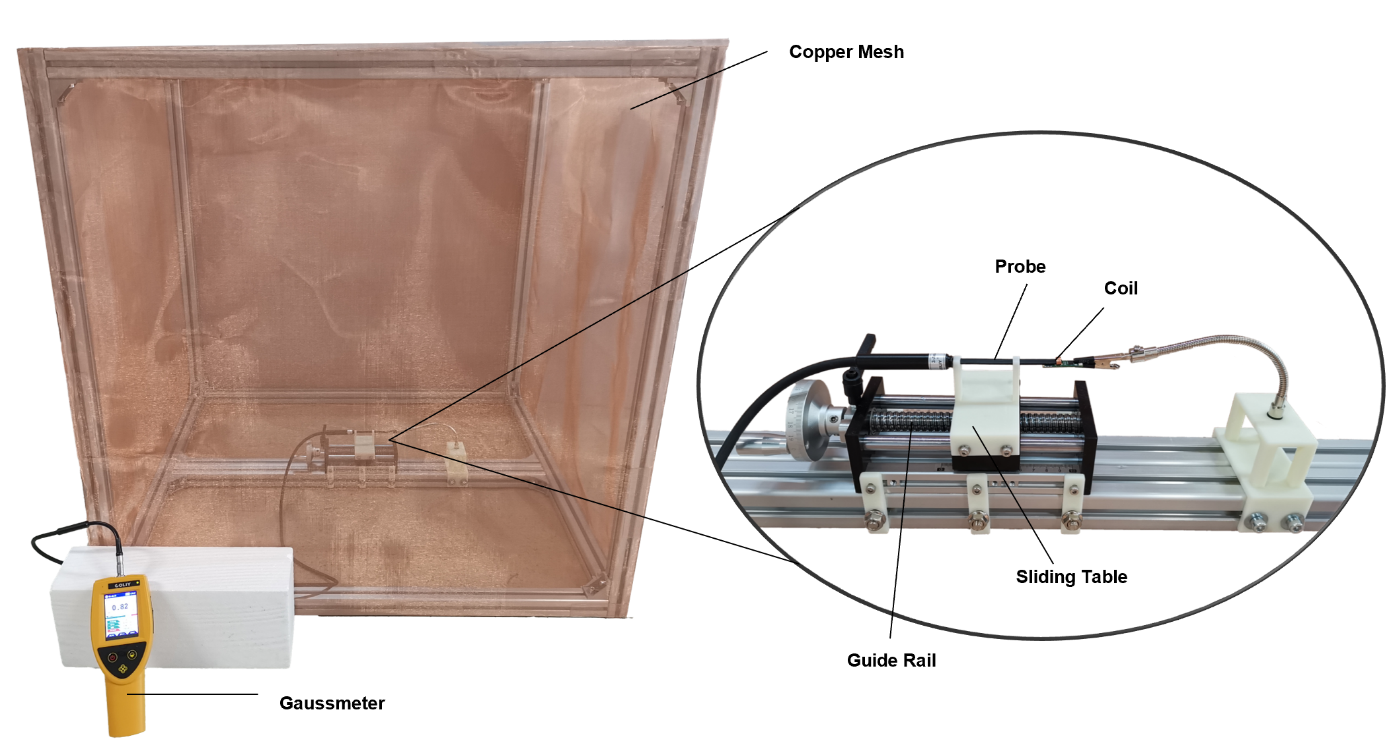


Fig. S11. Physical diagram of the coil magnetic field verification device. The figure in the circle shows a partial enlargement of the main experimental platform.


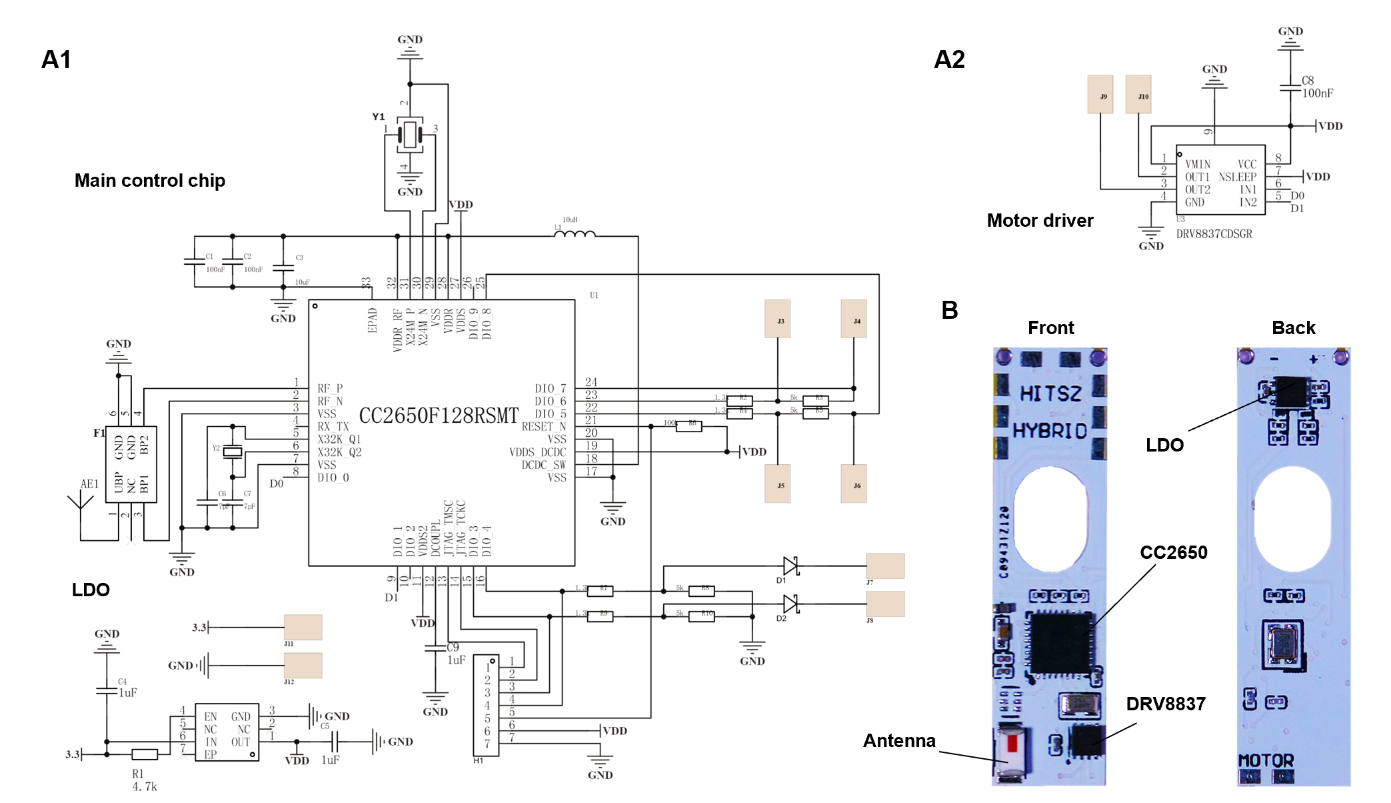


Fig. S12. Principle of remote wireless control of biohybrid locusts. (A1) and (A2) Schematic diagram of the PCB. (B) Physical diagram of the PCB.


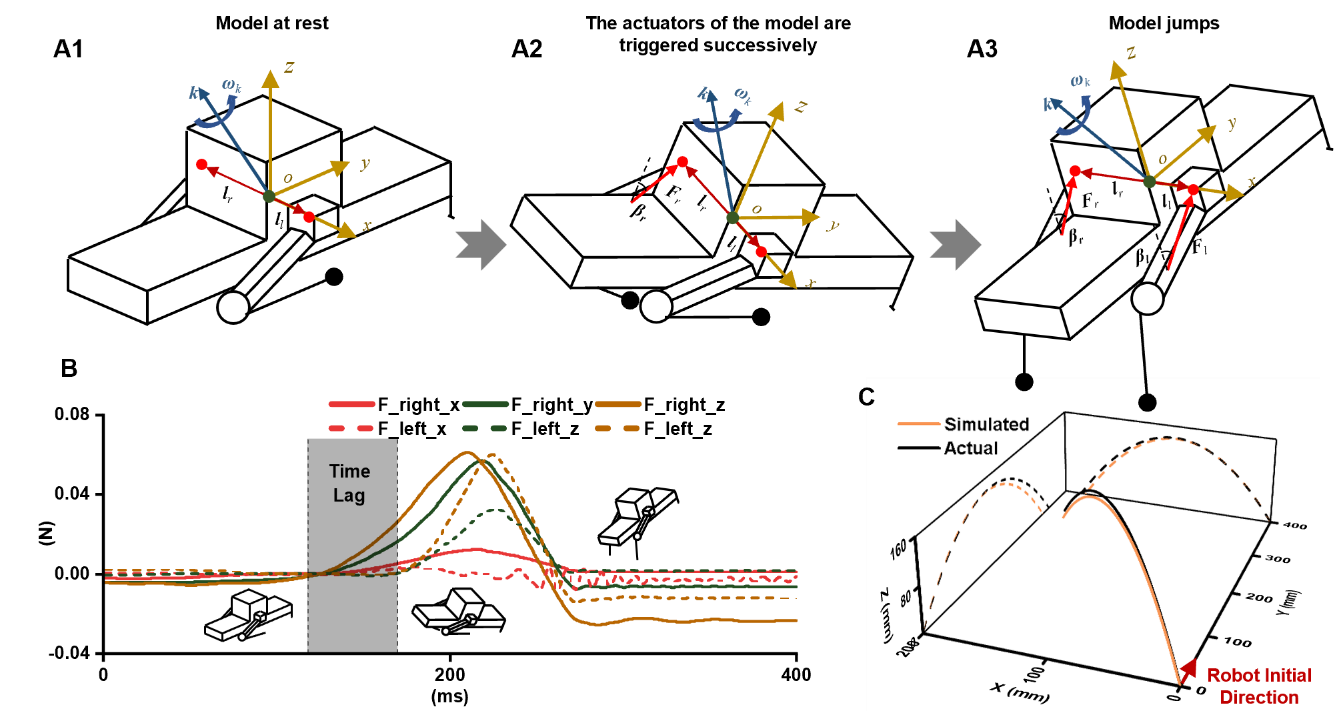


Fig. S13. Kinetic modelling of biohybrid locusts. (A1), (A2) and (A3) Schematic diagram of the dynamic model of the biohybrid locust. (B) Representative force curves of two bio-actuators in a steering jump. Time lag is labeled in shaded area. (C) The simulation trajectory based on the dynamic model is highly identical to the actual trajectory. Curves in space represent actual (simulated) jump trajectories, and curves in the plane represent projections of jump trajectories onto the corresponding planes.


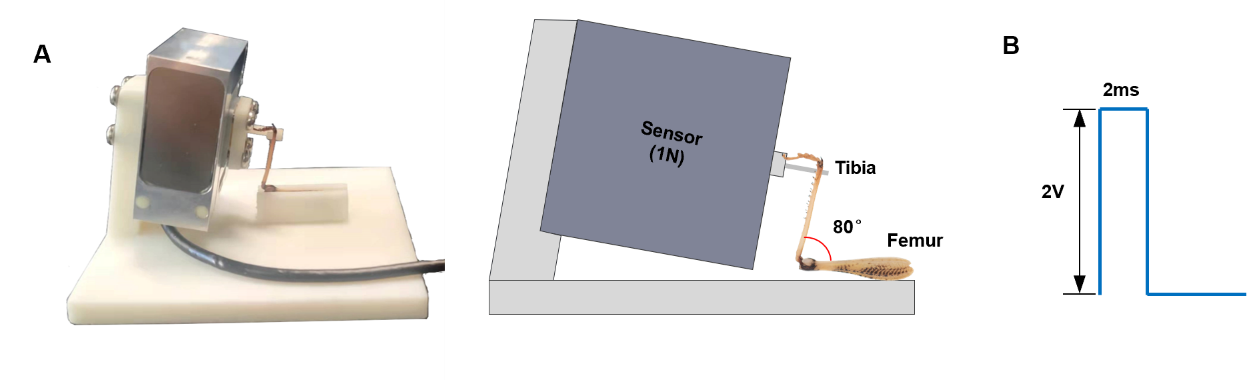


Fig. S14. Measurement of muscle isotonicity of locust isolated hindlegs. (A) Physical and schematic diagrams of the isolated hindleg muscle isotonic measurement device. (B) Electrical stimulation waveform.

Table S1. Comparison of Biohybrid locust with small jumping robotic systems and animals.

|  | **Name** | **Mass (g)** | **Energy per unit mass (mJ/g)** | **COT (mJ/g·m)** |
| --- | --- | --- | --- | --- |
| **Animals** | Ant [6] | 0.01 | 1.15 | 5.17 |
|  | Locust [2] | 2 | 3.06 | 5.1 |
|  | Spider [7] | 0.15 | 2.45 | 6.13 |
| **Robots** | Tribot [8] | 9.7 | 1.032 | 11.68 |
|  | Moobot [9] | 6 | 2.45 | 8.17 |
|  | Salto-1P [10] | 111 | 5.71 | 17.51 |
|  | Jump–Crawler [11] | 99.1 | 7.87 | 39.35 |
|  | Self-Recoverier [12] | 9.8 | 7.65 | 15.3 |
|  | Steerable Jumper [13] | 13.6 | 6.23 | 15.59 |
|  | Soft Robot [14] | 1.1 | 0.46 | 5.1 |
|  | Soft Jumper [15] | 0.5 | 0.83 | 10.41 |
|  | Differential Jumper [16] | 30 | 2.13 | 7.11 |
|  | JumpRoACH [17] | 99 | 9.56 | 6.38 |
|  | MSU [18] | 23.5 | 9.11 | 10.15 |
|  | Locust-bot [19] | 22.6 | 32.16 | 10.72 |
|  | Jumping-crawling robot [20] | 59.4 | 15.82 | 26.36 |
|  | 7g jumper [21] | 7 | 13.63 | 27.27 |
|  | locust-inspired jumper [22] | 25 | 24.75 | 24.75 |
|  | Continuous Jumper [23] | 120 | 24.5 | 6.13 |
|  | Jump-Runner [24] | 25 | 14.15 | 47.17 |
| **This Work** | Biohybrid locust | 2 | 2.94 | 4.9 |

Table S2. Curve parameters of flexor isotonic model.

|  | PF  (mN) | FTI  (mN·ms) | HRT  (ms) | LRT  (ms) | TPT  (ms) |
| --- | --- | --- | --- | --- | --- |
| Sample 1 | 6.64 | 150.52 | 9 | 14 | 10 |
| Sample 2 | 6.78 | 151.10 | 9 | 11 | 12 |
| Sample 3 | 6.82 | 154.32 | 9 | 12 | 12 |
| Sample 4 | 6.15 | 155.73 | 9 | 17 | 12 |
| Sample 5 | 5.89 | 148.94 | 10 | 18 | 12 |
| Sample 6 | 5.70 | 149.52 | 7 | 19 | 13 |
| Average value | 6.38 | 151.69 | 8.83 | 15.17 | 11.83 |
| second-order model response | 5.69 | 143.17 | 11 | 12 | 11 |
| Third-order model response | 6.24 | 153.26 | 9 | 13 | 13 |

Table S3. Curve parameters of extensor isotonic model.

|  | PF  (mN) | FTI  (mN·ms) | HRT  (ms) | LRT  (ms) | TPT  (ms) |
| --- | --- | --- | --- | --- | --- |
| Sample 1 | 84.00 | 7368.18 | 12 | 38 | 0.043 |
| Sample 2 | 82.20 | 6906.96 | 13 | 33 | 0.043 |
| Sample 3 | 76.30 | 6198.90 | 15 | 32 | 0.040 |
| Sample 4 | 83.86 | 7774.78 | 16 | 36 | 0.043 |
| Sample 5 | 78.84 | 6899.72 | 15 | 34 | 0.042 |
| Sample 6 | 79.70 | 6999.58 | 16 | 36 | 0.044 |
| Average value | 80.82 | 7024.69 | 14.5 | 34.83 | 0.043 |
| Fifth-order model response 1 | 85.08 | 7109.99 | 29 | 30 | 0.045 |
| Fifth-order model response 2 | 80.44 | 7096.69 | 23 | 27 | 0.046 |

Table S4. Extensor isotonic contraction model coefficient, 95 % confidence boundary and goodness of fit.

| Frequencies (Hz) | | 30 | 50 | 70 | 80 | 90 |
| --- | --- | --- | --- | --- | --- | --- |
| p1 | Value | -0.2639 | -0.4123 | -0.447 | -0.408 | -0.4533 |
|  | Upper limit | -0.2919 | -0.4644 | -0.5016 | -0.4509 | -0.504 |
|  | Lower limit | -0.236 | -0.3603 | -0.3924 | -0.3651 | -0.4026 |
| p2 | Value | 0.514 | 0.8248 | 0.911 | 0.8384 | 0.9391 |
|  | Upper limit | 0.4644 | 0.7286 | 0.8094 | 0.7596 | 0.8446 |
|  | Lower limit | 0.5636 | 0.921 | 1.0127 | 0.9172 | 1.0337 |
| p3 | Value | -0.3344 | -0.5512 | -0.6216 | -0.5767 | -0.652 |
|  | Upper limit | -0.3635 | -0.6103 | -0.6844 | -0.6247 | -0.7106 |
|  | Lower limit | -0.3052 | -0.4921 | -0.5588 | -0.5286 | -0.5934 |
| p4 | Value | 0.074 | 0.1252 | 0.1443 | 0.1347 | 0.154 |
|  | Upper limit | 0.0683 | 0.113 | 0.1313 | 0.1249 | 0.1418 |
|  | Lower limit | 0.0797 | 0.1374 | 0.1573 | 0.1445 | 0.1662 |
| p5 | Value | -0.0006 | -0.0011 | -0.0012 | -0.001 | -0.0012 |
|  | Upper limit | -0.0007 | -0.0013 | -0.0014 | -0.0011 | -0.0014 |
|  | Lower limit | -0.0005 | -0.001 | -0.0011 | -0.0009 | -0.0011 |
| q1 | Value | -0.7104 | -0.6727 | -0.7297 | -0.7774 | -0.7655 |
|  | Upper limit | -0.7307 | -0.7024 | -0.7558 | -0.7959 | -0.7882 |
|  | Lower limit | -0.6902 | -0.643 | -0.7035 | -0.7589 | -0.7427 |
| q2 | Value | 0.0379 | -0.016 | 0.0089 | 0.0509 | 0.0248 |
|  | Upper limit | 0.0174 | -0.0476 | -0.0206 | 0.0298 | -0.0013 |
|  | Lower limit | 0.0583 | 0.0156 | 0.0383 | 0.0719 | 0.051 |
| q3 | Value | 0.0424 | 0.0626 | 0.064 | 0.0541 | 0.0649 |
|  | Upper limit | 0.0371 | 0.0539 | 0.0555 | 0.048 | 0.0572 |
|  | Lower limit | 0.0477 | 0.0714 | 0.0725 | 0.0602 | 0.0725 |
| SSE | | 0.0057 | 0.008 | 0.0093 | 0.0082 | 0.0083 |
| R2 | | 0.9977 | 0.9978 | 0.9978 | 0.9981 | 0.9981 |
| DFE | | 394 | 394 | 394 | 394 | 394 |
| Adjusted R2 | | 0.9976 | 0.9977 | 0.9977 | 0.9981 | 0.9981 |
| RMSE | | 0.0038 | 0.0045 | 0.0049 | 0.0046 | 0.0046 |

Movie S1. Muscle contraction of the isolated locust hindlegs.

This movie shows the contractile action of the flexor (1/11 × real time), and the contractile action of the extensor (7/100 × real time) of the isolated hindleg under electrical stimulation.

Movie S2. Kicking process of the isolated locust hindlegs.

This movie first shows the kicking action of an isolated hindleg excited by an electrical stimulation sequence (1/80 × real time). Then it shows the kicking process (1/80 × real time) of the double isolated hindlegs under electrical stimulation at different time lags (0 ms, 10 ms, 20 ms, 30 ms, 40 ms, and 50 ms), respectively.

Movie S3. Jumping process of biohybrid locusts.

This movie shows the whole process of jumping of the biohybrid locusts, including lifting the hindlegs, retracting the tibia, pressing down hindlegs and jumping from macro and close-up perspectives respectively, as well as the process of jumping of the biohybrid locusts under high-speed camera (1/20 × real time).

Movie S4. Jumping performance of biohybrid locusts.

This movie shows the jumping height and jumping distance of a biohybrid locust during a single jump.

Movie S5. Steering jumps via asynchronous kicks of locusts and robots.

This movie shows the process of steer jumping via asynchronous kicking of locusts and robots respectively (1/20 × real time).

Movie S6. Continual jumping of biohybrid locusts.

This movie begins by demonstrating the self-righting ability of the biohybrid locust. The biohybrid locust righted itself by oscillating its artificial joints to provide additional angular momentum. Next, the movie demonstrates the untethered continual jumping of the biohybrid locust. The biohybrid locust quickly righted itself after one jump without external assistance, followed by the next jump.

Movie S7. Demonstration of stair climbing by biohybrid locusts.

This movie shows a biohybrid locust jumping continually in order to climb a staircase. One step is 40cm long and 10cm high.

**References**

[1] Ache JM, Matheson T. Passive Joint Forces Are Tuned to Limb Use in Insects and Drive Movements without Motor Activity. *Current Biology*. 2013;23(15):1418-1426. doi:10.1016/j.cub.2013.06.024

[2] Burrows M. The Neurobiology of an Insect Brain | Oxford Academic. Published online 1996. Accessed August 16, 2024. 10.1093/acprof:oso/9780198523444.001.0001

[3] Wilson E, Rustighi E, Mace BR, Newland PL. Isometric force generated by locust skeletal muscle: responses to single stimuli. *Biol Cybern*. 2010;102(6):503-511. doi:10.1007/s00422-010-0382-x

[4] Wilson E, Rustighi E, Mace BR, Newland PL. Modelling the isometric force response to multiple pulse stimuli in locust skeletal muscle. *Biol Cybern*. 2011;104(1-2):121-136. doi:10.1007/s00422-011-0423-0

[5] Kohut NJ, Birkmeyer PM, Peterson KC, Fearing RS. Maneuverability and mobility in palm-sized legged robots. In: George T, Islam MS, Dutta A, eds. *SPIE Defense, Security, and Sensing*. ; 2012:83731I. doi:10.1117/12.917874

[6] Patek SN, Baio JE, Fisher BL, Suarez AV. Multifunctionality and mechanical origins: Ballistic jaw propulsion in trap-jaw ants. *Proc Natl Acad Sci USA*. 2006;103(34):12787-12792. doi:10.1073/pnas.0604290103

[7] Nabawy MRA, Sivalingam G, Garwood RJ, Crowther WJ, Sellers WI. Energy and time optimal trajectories in exploratory jumps of the spider Phidippus regius. *Sci Rep*. 2018;8(1):7142. doi:10.1038/s41598-018-25227-9

[8] Zhakypov Z, Mori K, Hosoda K, Paik J. Designing minimal and scalable insect-inspired multi-locomotion millirobots. *Nature*. 2019;571(7765):381-386. doi:10.1038/s41586-019-1388-8

[9] Tang L, Li Y, Li B. Moobot: A Miniature Origami Omnidirectional Jumping Robot With High Trajectory Accuracy. *IEEE Trans Ind Electron*. 2024;71(6):6032-6040. doi:10.1109/TIE.2023.3294629

[10] Yim JK, Singh BRP, Wang EK, Featherstone R, Fearing RS. Precision Robotic Leaping and Landing Using Stance-Phase Balance. *IEEE Robot Autom Lett*. 2020;5(2):3422-3429. doi:10.1109/LRA.2020.2976597

[11] Chae SH, Baek SM, Lee J, Cho KJ. Agile and Energy-Efficient Jumping–Crawling Robot Through Rapid Transition of Locomotion and Enhanced Jumping Height Adjustment. *IEEE/ASME Trans Mechatron*. 2022;27(6):5890-5901. doi:10.1109/TMECH.2022.3190673

[12] Kovac M, Schlegel M, Zufferey JC, Floreano D. A miniature jumping robot with self-recovery capabilities. In: *2009 IEEE/RSJ International Conference on Intelligent Robots and Systems*. IEEE; 2009:583-588. doi:10.1109/IROS.2009.5354005

[13] Kovač M, Schlegel M, Zufferey JC, Floreano D. Steerable miniature jumping robot. *Auton Robot*. 2010;28(3):295-306. doi:10.1007/s10514-009-9173-4

[14] Chen R, Yuan Z, Guo J, Bai L, Zhu X, Liu F, Pu H, Xin L, Peng Y, Luo J, Wen L, Sun Y. Legless soft robots capable of rapid, continuous, and steered jumping. *Nat Commun*. 2021;12(1):7028. doi:10.1038/s41467-021-27265-w

[15] Tang D, Zhang C, Pan C, Hu H, Sun H, Dai H, Fu J, Majidi C, Zhao P. Bistable soft jumper capable of fast response and high takeoff velocity. *Sci Robot*. 2024;9(93):eadm8484. doi:10.1126/scirobotics.adm8484

[16] Bandyopadhyay T, Von-Richter K, Pallaud MA, Elfes A. Differential jumping: A novel mode for micro-robot navigation. In: *2016 IEEE International Conference on Robotics and Automation (ICRA)*. IEEE; 2016:3813-3818. doi:10.1109/ICRA.2016.7487570

[17] Jung GP, Casarez CS, Lee J, Baek SM, Yim SJ, Chae SH, Fearing RS, Cho KJ. JumpRoACH: A Trajectory-Adjustable Integrated Jumping–Crawling Robot. *IEEE/ASME Trans Mechatron*. 2019;24(3):947-958. doi:10.1109/TMECH.2019.2907743

[18] Zhao J, Xu J, Gao B, Xi N, Cintron FJ, Mutka MW, Xiao L. MSU Jumper: A Single-Motor-Actuated Miniature Steerable Jumping Robot. *IEEE Trans Robot*. 2013;29(3):602-614. doi:10.1109/TRO.2013.2249371

[19] Zaitsev V, Gvirsman O, Ben Hanan U, Weiss A, Ayali A, Kosa G. Locust-inspired miniature jumping robot. In: *2015 IEEE/RSJ International Conference on Intelligent Robots and Systems (IROS)*. IEEE; 2015:553-558. doi:10.1109/IROS.2015.7353426

[20] Jung GP, Casarez CS, Jung SP, Fearing RS, Cho KJ. An integrated jumping-crawling robot using height-adjustable jumping module. In: *2016 IEEE International Conference on Robotics and Automation (ICRA)*. IEEE; 2016:4680-4685. doi:10.1109/ICRA.2016.7487668

[21] Kovac M, Fuchs M, Guignard A, Zufferey JC, Floreano D. A miniature 7g jumping robot. In: *2008 IEEE International Conference on Robotics and Automation*. IEEE; 2008:373-378. doi:10.1109/ROBOT.2008.4543236

[22] Beck A, Zaitsev V, Hanan UB, Kosa G, Ayali A, Weiss A. Jump stabilization and landing control by wing-spreading of a locust-inspired jumper. *Bioinspir Biomim*. 2017;12(6):066006. doi:10.1088/1748-3190/aa8ceb

[23] Yang Y, Feng Z, Ma S, Tang L, Jin C, Li Y. The Continuous Jump Control of a Locust-Inspired Robot With Omnidirectional Trajectory Adjustment. *IEEE Robot Autom Lett*. 2024;9(3):2040-2047. doi:10.1109/LRA.2024.3349808

[24] Zhao J, Yan W, Xi N, Mutka MW, Xiao L. A miniature 25 grams running and jumping robot. In: *2014 IEEE International Conference on Robotics and Automation (ICRA)*. IEEE; 2014:5115-5120. doi:10.1109/ICRA.2014.6907609
